# A Complex Evolutionary History in a Remote Archipelago: Phylogeography and Morphometrics of the Hawaiian Endemic *Ligia* Isopods

**DOI:** 10.1371/journal.pone.0085199

**Published:** 2013-12-30

**Authors:** Carlos A. Santamaria, Mariana Mateos, Stefano Taiti, Thomas J. DeWitt, Luis A. Hurtado

**Affiliations:** 1 Department of Wildlife and Fisheries Sciences, Texas A&M University, College Station, Texas, United States of America; 2 Department of Biological Sciences, Sam Houston State University, Huntsville, Texas, United States of America; 3 Istituto per lo Studio degli Ecosistemi, CNR, Florence, Italy; George Washington University, United States of America

## Abstract

Compared to the striking diversification and levels of endemism observed in many terrestrial groups within the Hawaiian Archipelago, marine invertebrates exhibit remarkably lower rates of endemism and diversification. Supralittoral invertebrates restricted to specific coastal patchy habitats, however, have the potential for high levels of allopatric diversification. This is the case of *Ligia* isopods endemic to the Hawaiian Archipelago, which most likely arose from a rocky supralittoral ancestor that colonized the archipelago via rafting, and diversified into rocky supralittoral and inland lineages. A previous study on populations of this isopod from Oʻahu and Kauaʻi revealed high levels of allopatric differentiation, and suggested inter-island historical dispersal events have been rare. To gain a better understanding on the diversity and evolution of this group, we expanded prior phylogeographic work by incorporating populations from unsampled main Hawaiian Islands (Maui, Molokaʻi, Lanaʻi, and Hawaiʻi), increasing the number of gene markers (four mitochondrial and two nuclear genes), and conducting Maximum likelihood and Bayesian phylogenetic analyses. Our study revealed new lineages and expanded the distribution range of several lineages. The phylogeographic patterns of *Ligia* in the study area are complex, with Hawaiʻi, Oʻahu, and the Maui-Nui islands sharing major lineages, implying multiple inter-island historical dispersal events. In contrast, the oldest and most geographically distant of the major islands (Kauaʻi) shares no lineages with the other islands. Our results did not support the monophyly of all the supralittoral lineages (currently grouped into *L. hawaiensis*), or the monophyly of the terrestrial lineages (currently grouped into *L. perkinsi*), implying more than one evolutionary transition between coastal and inland forms. Geometric-morphometric analyses of three supralittoral clades revealed significant body shape differences among them. A taxonomic revision of Hawaiian *Ligia* is warranted. Our results are relevant for the protection of biodiversity found in an environment subject to high pressure from disturbances.

## Introduction

The Hawaiian Islands are well known for their rich biodiversity and high rate of endemism [1]. Their remoteness, representing the world’s most isolated major archipelago, along with the progressive formation of these islands, are considered crucial for the striking diversification observed in several Hawaiian terrestrial organisms, which include: the Hawaiian *Drosophila* [2-4], the silversword alliance [5], succineid land snails [6,7], honeycreeper birds [8,9], and others [10-13]. In contrast, endemism in Hawaiian marine invertebrates is strikingly lower than that in Hawaiian terrestrial organisms [14], and with the exception of intertidal *Cellana* limpets [15], there are no documented marine radiations within the Hawaiian Archipelago. Such disparity suggests that opportunities for allopatric differentiation within the archipelago have been very limited in the marine realm, while abundant in the terrestrial realm. Populations of non-vagile invertebrates endemic to specific coastal patchy habitats located at the interface between sea and land, however, have the potential to be highly isolated and, thus, show elevated levels of allopatric differentiation [16].

The Hawaiian endemic shrimp *Halocaridinia rubra*, which inhabits anchialine coastal pools, shows evidence of within- and between- island divergence and is comprised of multiple highly divergent lineages [17,18]. An additional interesting case of diversification in a coastal patchy habitat of the archipelago is that of oniscidean isopods in the genus *Ligia*, which most likely arose from a rocky supralittoral ancestor that arrived to the Hawaiian Archipelago via rafting, and diversified into rocky supralittoral and inland (hereafter, terrestrial) lineages. High levels of genetic differentiation were detected among populations of Hawaiian *Ligia* from different localities in the islands of Kauaʻi and Oʻahu [19], but populations from other islands have not been studied. Therefore, further phylogeographic analyses of Hawaiian *Ligia* including populations from previously unsampled islands are needed to better understand the biodiversity and evolution of this group.

The genus *Ligia* has a worldwide distribution and includes 42 currently recognized species [20-26]; most of which are restricted to rocky supralittoral areas [19,27]. Eight species, however, are strictly terrestrial, inhabiting montane habitats of tropical regions [19,22], and are believed to derive from supralittoral forms [28]. Supralittoral *Ligia* exhibit morphological, physiological, and behavioral characteristics that are intermediate between ancestral marine and fully terrestrial isopods [29]. Some of the biological characteristics of supralittoral *Ligia* confer them extremely low vagility [30], such as: direct development (lack a planktonic larval phase, as all peracarids); active avoidance of the open sea (they remain in the area between the exposed intertidal and the supralittoral); extremely low desiccation tolerance (a reason for which they stay close to the water line and are most active at night); limited motility underwater and on sandy shores (rendering them highly vulnerable to predators in these environments).

The biology of supralittoral *Ligia* severely constrains the movement of these isopods outside the rocky beaches they occupy, effectively isolating populations. This is reflected in the striking radiations of supralittoral *Ligia* reported in different regions of the world, with extraordinarily high levels of allopatric genetic differentiation, even between localities separated by few kilometers ([19,30-32] and unpublished data). These observations, as well as those by Hurtado et al. [16], challenge earlier suggestions that supralittoral isopods are highly dispersive species, based on their common presence in beaches around the world [33]. Phylogeographic patterns of *Ligia* in different regions have been shaped by past tectonic events [30], environmental factors, such as sea surface temperature [31], as well as oceanic dispersal events, probably through rafting (unpublished data).

Two endemic species of *Ligia* are currently recognized in the Hawaiian Islands [34]: the rocky supralittoral *Ligia hawaiensis* (Dana 1853) and the terrestrial montane *Ligia perkinsi* (Dollfus 1900). A third species, the cosmopolitan introduced *Ligia exotica* (Roux 1828), is reported from littoral man-made substrate [19,35]. The supralittoral species *L. hawaiensis* occurs in rocky intertidal habitats throughout the archipelago [27,34,36], whereas the terrestrial species *L. perkinsi* is known from high altitude (300–1,500 m above sea level) wet forests on the islands of Kauaʻi, Oʻahu, and Hawaiʻi [34]; although the last report of *L. perkinsi* in Hawaiʻi was in 1896 [19].

Taiti et al. [19] investigated whether the origin of terrestriality in *L. perkinsi* populations from the Hawaiian islands of Kauaʻi and Oʻahu occurred as a single event (i.e., *L. perkinsi* from both islands constitute a monophyletic group sister to a lineage of *L. hawaiensis*) or as two independent events, one in each island (e.g. two clades each showing reciprocal monophyly of *L. hawaiensis* and *L. perkinsi* from the same island). They conducted Maximum Parsimony phylogenetic analyses with sequences of two mitochondrial genes (COI and 16S rDNA), and included individuals of *L. perkinsi* and *L. hawaiensis* from Kauaʻi and Oʻahu. They obtained strong support for the monophyly of the *Ligia* lineages endemic to the Hawaiian Archipelago (also observed in [30]), which were divided into three main clades: one comprised of the *L. perkinsi* from Kauaʻi; another clade comprised of *L. perkinsi* from Oʻahu; and a third clade comprised of *L. hawaiensis* individuals. The *L. hawaiensis* clade was divided into three lineages: one comprised of the Kauaʻi individuals and the other two comprised of the Oʻahu individuals. The two main *L. perkinsi* clades were paraphyletic (*L. perkinsi* from Oʻahu was sister to a clade of *L. perkinsi* from Kauaʻi + *L. hawaiensis*), thus, the results were inconclusive as to whether a single or two origins of terrestriality occurred. In addition, they observed high divergences among populations of *L. hawaiensis*, implying long-standing isolation among them, and with phylogeographic patterns suggesting inter-island dispersal events have been rare throughout the history of the Hawaiian endemic *Ligia* lineages [19].

Herein, we expanded on Taiti et al.’s [19] previous work by incorporating populations from previously unsampled main Hawaiian Islands (i.e., Maui, Molokaʻi, Lanaʻi, and Hawaiʻi), increasing the number of gene markers, which include nuclear genes, and applying more current phylogenetic approaches. Sampling across all main Hawaiian Islands enables a better understanding on the diversity and evolution of endemic Hawaiian *Ligia*. Specifically, we asked: (1) whether the younger islands (i.e., Maui, Lanaʻi, and Molokaʻi, all with an age ~ 1.3 My, and Hawaiʻi with an age of 0.4 My; ages from [37]) harbor highly divergent *L. hawaiensis* lineages, as observed in the older islands (i.e., Kauaʻi [5.1 My] and Oʻahu [3.7 My]); (2) whether evolution of *Ligia* in the Hawaiian Islands followed a pattern consistent with the progression rule (i.e., lineages from older islands are basal to those from younger islands), a back dispersal pattern (lineages from younger islands colonized the older islands), or an unresolved and/or highly stochastic pattern (indicative of a complex evolutionary history, probably with frequent inter-island dispersal); and (3) whether the new data shed light on the origin of terrestriality in Kauaʻi and Oʻahu (i.e., a single origin or independent origins in each island). Lastly, we incorporated geometric-morphometric analyses to test for differences among three highly divergent supralittoral lineages and determine whether these methods can be used for their discrimination.

## Materials and Methods

### Sampling

**Figure 1:**
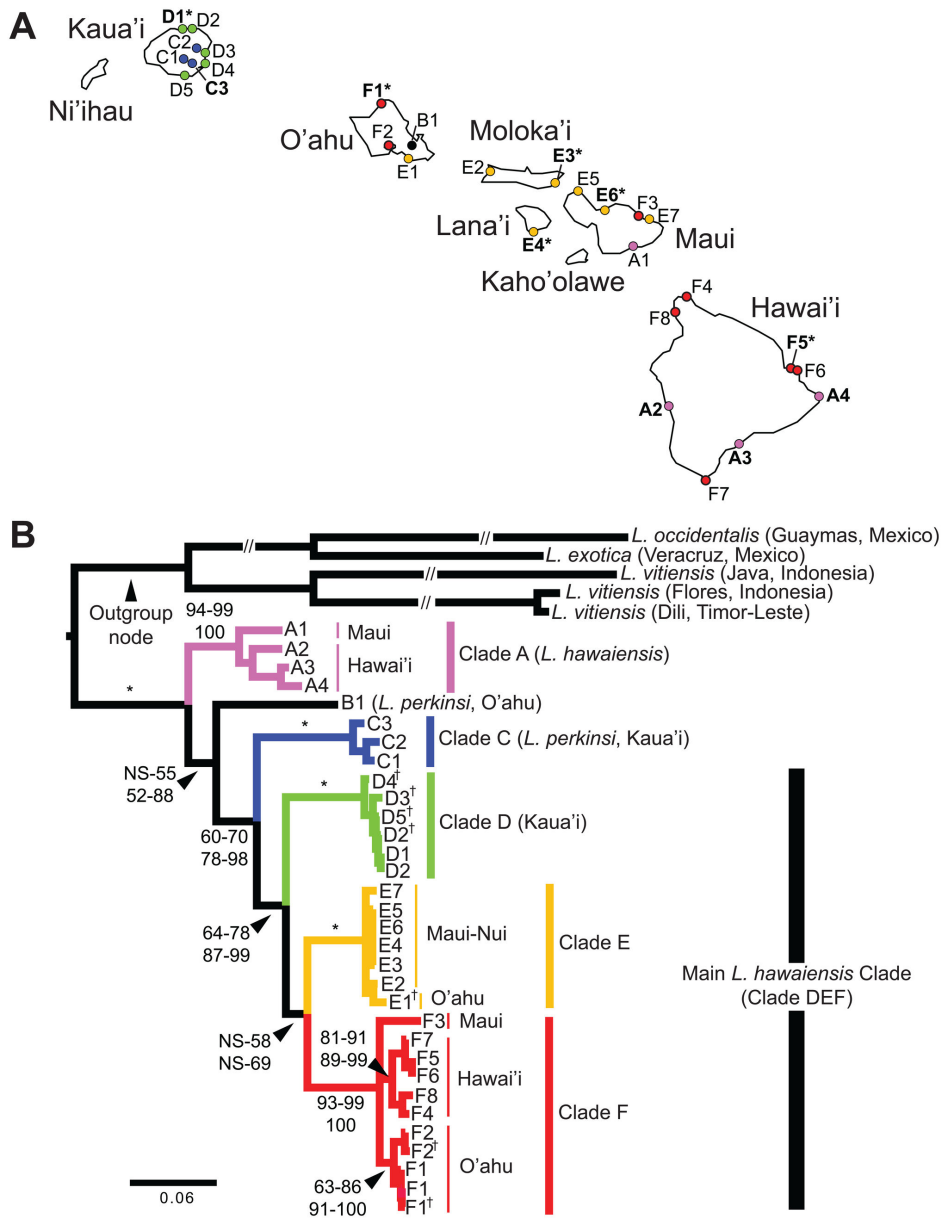
Sampled localities (A) and inferred phylogeny (B) of *Ligia* lineages endemic to the Hawaiian Archipelago. Color-coding and labels correspond between panels and with other figures and tables. Detailed information for each locality is presented in Table S1. A. Sampled localities for supralittoral *L. hawaiensis*: D1-Kapua'a Beach Park, D2-Kauapea Beach, D3-Kapa'a, D4-Lihu'e, D5-Kukui'ula (Kaua’i Island); E1-Ala Wai Canal, F1-Pupukea, F2-Pouhala Marsh (O’ahu Island); E2-Papohaku Beach Park, E3-North of Puko'o; Lana’i: E4-Manele Bay (Moloka’I Island); A1-Waiopai, E5-Poelua Bay, E6-Spreckelsville, E7-Keanae, F3-Honomanu Bay (Maui Island); A2-Kealakukea Bay, A3-Pu'unalu Beach Park, A4-Isaac Hale Beach Park, F4-Keokea Beach, F5-Onekahakaha Beach Park, F6-Leleiwi Beach, F7-South Point, F8-Kapa'a State Park (Hawai’i Island). Sampled localities for terrestrial *L*. *perkinsi*: C1-Mt Kahili, C2-Makaleha Mts, C3-Haupu Range (Kaua’i Island); B1-Nu'uanu Pali (O’ahu Island). Boldfaced labels indicate localities used in the nuclear analyses. * indicates localities examined in the geometric morphometric analyses. B. Majority rule consensus tree (GTR +Γ model in RaxML; TreeBase http://purl.org/phylo/treebase/phylows/study/TB2:S14886) of the concatenated mitochondrial dataset of Ligia samples from the Hawaiian Archipelago and several outgroups. Numbers by nodes indicate the corresponding range of percent Bootstrap Support (BS; top) for Maximum likelihood; and Posterior Probabilities (PP; bottom) for Bayesian inference methods (clade support values for each analysis are shown in Table S4). Nodes receiving 100% for all methods are denoted with an *. NS: less than 50% node support. Samples examined by Taiti et al. [19] are marked with †.

Our molecular dataset included twenty-four *L. hawaiensis* populations (i.e., localities) from across the main Hawaiian Islands and four *L. perkinsi* populations from Kauaʻi and Oʻahu (Figure 1A, Table S1). The collection of these specimens did not require a permit from the Department of Land and Natural Resources of Hawaii, and these isopods are not considered endangered or protected species. We also included publicly available sequences for *L. hawaiensis* and *L. perkinsi* (Table S1). We were unable to collect *L. hawaiensis* in the islands of Niʻihau and Kahoʻolawe, because they are private property and a state reserve, respectively. Populations were sampled by hand and preserved in 70–100% Ethanol. As outgroups, we included specimens from *Ligia vitiensis*, *Ligia occidentalis*, and *Ligia exotica*, as previous research [19,30] and preliminary analyses suggest they are the closest extant relatives to *Ligia* from the Hawaiian Archipelago.

### Molecular methods

We extracted total genomic DNA of *Ligia* individuals from pleopods/legs using the DNEasy Blood & Tissue kit (Qiagen Inc.), following standard protocol instructions. We PCR-amplified a 710-bp fragment of the Cytochrome Oxidase I (COI) mitochondrial (mt) gene for 1–10 individuals per population (Dataset S1) using the primers and conditions published by Folmer et al. [38]. A subset of these individuals (essentially one individual per population; see Figure S1) was then amplified and sequenced for three additional mitochondrial genes using previously published primers and conditions: ~490-bp of the 16S rDNA gene (primers 16Sar/16Sbr; [39]); ~495-bp of 12S rDNA (primers crust-12Sf/crust-12Sr; [40]); and a 361-bp fragment of the Cytochrome-b (Cytb) gene (primers 144F/151F and 270R/272R; [41]). We also amplified two nuclear genes for a subset of individuals (1–5 per population, see Figure S1): a ~1,000-bp region of the 28S rDNA gene (primers 28SA/28SB; [42]) and a ~710-bp region of the alpha-subunit of the Sodium Potassium ATPase (NaK; primers NaK forb/NaK rev 2; [43]). PCR-products were cleaned with a mixture of Exonuclease I (New England Biolabs) and Shrimp Alkaline Phosphatase (USB Scientific) and cycle sequenced at the University of Arizona Genetics Core (UAGC). We assembled sequences and removed primer regions using Sequencher 4.8 (Genecodes). None of the protein-coding sequences exhibited premature stop codons or frame shifts, suggesting they are not pseudogenes. 

### Sequence alignments and mitochondrial phylogenetic analyses

We aligned the ribosomal DNA gene fragments (i.e., 16S rDNA, 12S rDNA, and 28S rDNA) with the MAFFT algorithm [44] assuming the Q-INS-I strategy as implemented in the GUIDANCE server [45]. Because of the high divergence among lineages of *Ligia* (see Results), several regions of ambiguous alignment were observed for these genes. Therefore, we used the GUIDANCE server [46] to estimate confidence scores for each nucleotide position (100 independent alignments based on different bootstrap guide trees were conducted), and removed all positions with a confidence score below 1.00, as well as several positions for which alignments were considered ambiguous (Datasets S2 and S3). We estimated pairwise genetic distances with the Kimura-2-Parameter (K2P) correction (excluding ambiguous sites) in MEGA v5.05 [47] for the COI and the 16S rDNA gene fragments separately.

We determined the most appropriate model of DNA substitution for each mitochondrial gene fragment and the mitochondrial concatenated dataset from among 1,624 candidate models, by evaluating their corresponding likelihood scores on a fixed BioNJ-JC tree, under the Akaike Information Criterion (AIC), corrected AIC (AICc), and the Bayesian Information Criterion (BIC) (Table S2) using jModeltest v2.1.1 [48]. The chosen model was used in phylogenetic searches, with two general exceptions. First, when the selected model was not available in a particular software, we applied the next more complex model available (Table S3). Second, as the joint estimation of Γ and I parameters can be problematic (see RAxML manual; and pages 113-114 of [49]), we used the simpler Γ when the chosen model included both Γ and I parameters. We also implemented several partitioning schemes: (a) all positions within a single partition; (b) partitioned by gene; and (c) the best partitioning scheme according to the BIC implemented in PartitionFinder v1.0.0 [50]. We used the following parameters in PartitionFinder searches: branch lengths = linked; models = all; model selection = BIC; search = greedy; and *a priori* partitioning combining each gene and codon position.

We carried out maximum likelihood (ML) searches in RAxML v7.2.6 [51-53] and GARLI v2.0 [54]. RAxML consisted of 1,000 bootstrap replicates followed by a thorough ML search under the GTR +Γ model run under the Rapid Bootstrap Algorithm, whereas GARLI analyses consisted of 1,000 bootstrap replicates under the appropriate model of evolution identified by jModeltest. All other settings were as default. We calculated majority-rule consensus trees for each analysis with the SumTrees command of DendroPy v3.10.1 [55]. 

We carried out Bayesian phylogenetic reconstructions in MrBayes v3.1.2 [56,57] and Phycas v1.2.0 [58]. We implemented polytomy priors [59] in Phycas to alleviate the potential overestimation of posterior probabilities (i.e., “star-tree paradox”) known to affect Bayesian approaches [60]. We present the number of independent MCMC runs, chains, and generations in Table S3, with all other parameters as default. We determined if Bayesian analyses had reached stationarity by: (a) stable posterior probability values; (b) high correlation between the split frequencies of independent runs as implemented in AWTY [61]; (c) small and stable average standard deviation of the split frequencies of independent runs; (d) Potential Scale Reduction Factor close to 1; and (e) an Effective Sample Size (ESS) > 200 for the posterior probabilities, as evaluated in Tracer v1.5 [62]. Samples prior to stationarity were discarded as “burnin” (Table S3). To estimate the posterior probability for each node, we built majority-rule consensus trees of the stationary stage of each run using the SumTrees command [55].

### Nuclear gene analyses

Given the low variation levels observed in both nuclear genes amplified (see Results), we visualized relationships between nuclear alleles on networks constructed using the cladogram estimation algorithm of Templeton et al. [63] as implemented by TCS v1.21 [64]. We calculated the 95% most parsimoniously plausible branch connections between alleles, with all other settings as default. 

### Geometric-morphometric methods

**Figure 2:**
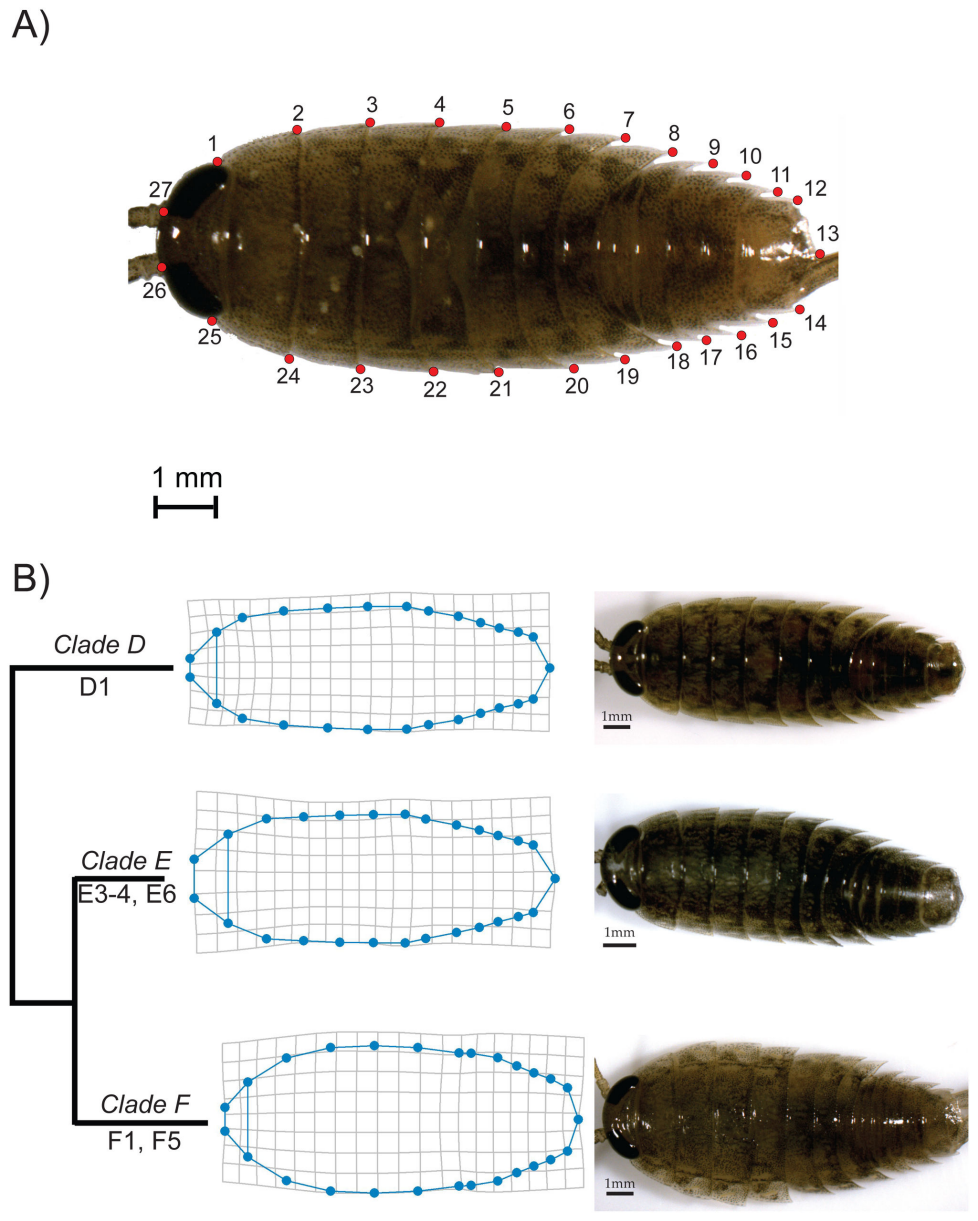
Landmarks (LMs) placement (A) and thin-plate-spline transformations (B) for *Ligia hawaiensis* (clades *D*, *E*, *F*). A. LMs 1 and 25 represent the posterior margin of the eyes at the body perimeter; LMs 2-11 and 15–24 are the posterior-most point of each segment; LMs 12 and 14 are the lateral posterior points of the pleotelson, whereas LM 13 is the posterior-most point of the pleotelson; LMs 26 and 27 correspond to the inner-most margin of the eyes. B. Thin-plate-spline transformations of LM positions are shown magnified (10 ×) to reach the natural extremes observed in the clades, which are also indicated with images for specimens with the highest probability of being correctly assigned to each clade.

We captured digital images of the dorsal side of *L. hawaiensis* specimens using QCapture v3.1.2 and an Olympus QColor3 digital camera attached to an Olympus SZ61 stereomicroscope. We removed all pereopods (i.e., legs) to ensure specimens laid flat. Dissected pereopods and pleopods were not used for morphometric comparisons. The male pereopods and pleopods, which are fundamental in distinguishing *Ligia* species, did not show distinct differences among the *L. hawaiensis* populations [19]. During dissections, we determined and noted the sex of each specimen by visually inspecting the endopod of the 2^nd^ pleopod. Sex was noted as either: male (M); gravid female (F); or juvenile/non-gravid female (J). We characterized body shape by digitizing 27 landmarks (LMs), using TpsDig v2.16 [65], on the periphery of *Ligia* bodies (Figure 2A). We included landmarks that capture taxonomically informative regions and can be noted unambiguously. For example, we placed landmarks on the medial and the lateral boundaries of the eyes at the body periphery. These landmarks capture the relative size of the eyes and the inter-ocular distance, characters used to distinguish *Ligia* species [19]. We characterized the relative width of body segments and overall body shape, also important in *Ligia* taxonomy [19,25,27,66,67], by placing landmarks on the lateral posterior tergite tips. Lastly, we captured the shape of the pleotelson, another trait used in *Ligia* taxonomy [19,25,68], by placing landmarks at its posterior tip and the lateral posterior points.

As the body plan of *Ligia* is bilaterally symmetric, all but the pleotelson tip LMs are anatomically homologous and should not be treated as independent in statistical analyses. As suggested by Zelditch et al. [69], we reflected and averaged homologous landmarks across the body midline as defined by the pleotelson tip and the midpoint between the medial eye LMs. Corrected landmarks were centered, scaled and rotated, to best align with the consensus, using the method of generalized least squares, and projected to a flat shape space using tpsRelw v1.49 [70]. We calculated principal components of aligned coordinates to yield orthogonal shape variables, retaining the minimum number of components needed to represent ≥ 95% of the overall variation. These principal components were taken as shape variables to test for differences in shape between lineages, sexes, and sizes. We used the centroid size (the summed square distances of landmarks from the centroid; [71]) as an estimate of body size. 

### Statistical analyses

We carried out full factorial MANCOVA analyses of shape variables as a function of lineage, sex, size, and all interactions, to discern the meaningful effects on body shape. When interaction terms were not significant, we removed them from the model, in a hierarchical manner, and repeated analyses. We estimated effect strengths by calculating partial eta squared values (η_p_^2^), which is the multivariate analog of *R* ^2^ in simple regression models [72]. We further explored differences between lineages with quadratic Discriminant Function Analyses (DFAs) with equal probability priors. To focus on these between-group differences, we first accounted for continuous predictors by conducting a preliminary MANCOVA and saving residual variation [73]. We used successful classification frequency as an intuitive metric of the power of morphological divergence to correctly assign an individual to its genetic lineage based solely on its morphology. All DFA results were validated using leave-one out cross validation (LOOCV). All statistical tests were carried out in JMP v9.0.1, except for η_p_^2^ values, which were calculated in Microsoft Excel® using the **E** & **H** matrix output from JMP. Lastly, we visualized shape differences between all main effects by producing thin-plate-spline transformations of LM positions in tpsRegr v1.37 [74], using canonical scores for the clade effect as the predictor variable block [75].

## Results

All new sequences produced in this study have been deposited in GenBank under accession Numbers KF546528-KF546728 (Table S1). Annotated alignments used in analyses are in Datasets S1–S4, TreeBase (http://purl.org/phylo/treebase/phylows/study/TB2:S14886), and DataDryad (doi:10.5061/dryad.5k56c).

### Mitochondrial phylogenetic results

The final concatenated mitochondrial dataset (mt) included 32 individuals from throughout the Hawaiian Archipelago, and five individuals from three *Ligia* species (*L. exotica*, *L. occidentalis*, and *L. vitiensis*) as outgroups (Dataset S2). We excluded 219 poorly aligned positions (16S rDNA: 113-bp; 12S rDNA: 106-bp), resulting in a final alignment of 1758 characters, 581 of which were parsimony informative (Table S2). Selection criteria in jModeltest did not agree on a single model for the dataset. The Bayesian Information Criterion (BIC) selected a model with three substitution rates (rate matrix: 012010, see jModeltest manual), variable nucleotide frequencies (+F), and both +I and +Γ parameters. Akaike Information Criteria strategies (AIC, AICc) selected a slightly more complex model consisting of four substitution rates (rate matrix: 012313, see jModeltest manual) and +F, +I, and +Γ parameters. Given the low weights observed for these models under the AIC and AICc (Table S2), and that the 95% confidence interval included the BIC selected model, we applied the latter in GARLI analyses. For all other software (e.g. RAxML, MrBayes), we applied the GTR +Γ model instead, as the chosen models cannot be implemented.

Phylogenetic relationships inferred from the mitochondrial dataset are shown in Figure 1B. Node support values for each analysis are provided in Table S4. The endemic Hawaiian Archipelago *Ligia* clade (i.e., ingroup) was highly supported: 100 Bootstrap Support (BS) and Posterior Probability (PP). Within this clade, we observed three basal lineages with divergences among them between 11.85 and 16.74% COI K2P (Table 1): (1) *Clade A* (lavender in Figure 1), which is a well-supported clade (94–99 BS; 100 PP) that included some *L. hawaiensis* populations from Maui and Hawaiʻi, and represents a new lineage that was not previously identified by Taiti et al. [19]; (2) *Lineage B* (black in Figure 1), which includes the *L. perkinsi* population from Oʻahu (B1), and was previously reported by Taiti et al. [19]; and (3) *Clade CDEF* (blue, green, orange, and red, respectively, in Figure 1; 60–70 BS and 78–98 PP), which contained all *L. perkinsi* samples from Kauaʻi (*Clade C*) and the rest of the *L. hawaiensis* samples from the archipelago (*Clade DEF*).

**Table 1:** Estimates of genetic divergence, as measured by Kimura-2-parameter distances, among the main *Ligia* lineages in the Hawaii Archipelago and outgroups.

	*Clade A*	*Clade B*	*Clade C*	*Clade D*	*Clade E*	*Clade F*	*L. exotica*	*L. occidentalis*	*L. vitiensis*
*Clade A*	**5.88 2.15-4.39**	14.52-14.81	11.85-13.79	13.57-16.74	13.96-14.63	14.36-15.96	23.77-25.04	21.17-21.18	19.76-24.54
*Clade B*	6.34-7.36	**N/A N/A**	14.22-14.87	15.14-15.81	15.75-16.65	13.04-14.39	24.11-24.11	22.96-22.96	19.08-24.54
*Clade C*	6.69-8.72	7.67-8.01	**0.88-2.51 0.30-1.22**	13.57-15.37	12.69-14.24	12.03-14.44	24.62-25.13	20.93-21.90	19.26-25.23
*Clade D*	7.02-7.72	5.65-6.31	4.36-5.33	**0.00-1.60 0.00-0.91**	10.53-12.93	12.20-13.99	23.66-24.42	23.33-23.83	20.52-26.53
*Clade E*	7.04-9.49	6.32-6.67	5.66-6.68	3.72-5.36	**0.00-2.51 0.00-1.84**	10.51-12.91	23.93-24.73	23.08-23.58	22.16-26.45
*Clade F*	7.74-10.22	8.39-10.17	5.66-6.68	5.36-7.72	4.07-6.40	**0.18-5.30 0.00-1.85**	22.17-23.02	22.12-23.60	20.47-24.57
*L. exotica*	14.89-16.04	16.41-16.41	12.88-13.25	14.04-14.81	14.83-15.25	15.32-16.12	**N/A N/A**	22.65-22.65	22.82-24.34
*L. occidentalis*	19.02-20.66	19.88-19.88	18.58-19.85	18.26-19.05	20.69-21.12	19.09-20.35	13.00-13.00	**N/A N/A**	22.65-23.32
*L. vitiensis*	19.05-21.90	18.37-20.20	17.77-20.59	16.01-17.77	15.19-18.55	17.92-19.16	21.20-23.90	24.02-25.24	**0.30-17.89 3.06-23.02**

Above matrix: COI gene distances; below matrix: 16S rDNA gene distances. Diagonal (in bold) indicate within-clade distances (upper values: COI; below: 16S rDNA).

Within *Clade A*, we detected three divergent lineages (maximum COI K2P divergence = 5.88%; Table 1): (1) one found in a single Maui population (A1); (2) another in a single population from western Hawaiʻi (A2); and (3) the last in eastern Hawaiʻi (A3, A4). The analyses suggest the Maui lineage represents the most basal split within *Clade A* and that lineages from Hawaiʻi form a monophyletic group; support for this relationship, however, was variable (66–85 BS; < 65 PP).

Within *Clade CDEF* we observed a basal split between *Clade C* (blue in Figure 1; 100 BS; 100 PP), which contained all *L. perkinsi* localities from Kauaʻi (C1-C3), and the *L. hawaiensis* Clade *DEF* (64–78 BS; 87–99 PP). Maximum COI K2P divergence within *Clade C* was 2.51% (Table 1); and this lineage was previously identified by Taiti et al. [19]. Within *Clade DEF*, relationships among clades *D*, *E*, and *F* were unresolved. *Clade D* (light green in Figure 1; 100 BS and 100 PP) includes four *L. hawaiensis* localities from Kauaʻi (D2-D5) previously sampled by Taiti et al. [19], and one newly sampled in this study (D1). Maximum within-clade COI K2P divergence in *Clade D* was 1.60%. A member of *Clade E* (orange in Figure 1; 100 BS and 100 PP) was previously sampled in Taiti et al. [19] from Oʻahu (E1); we discovered that the distribution of this clade extends to Molokaʻi (E2, E3), Lanaʻi (E4), and Maui (E5-E7). Maximum within-clade COI K2P divergence in *Clade E* was 2.50%. Lastly, *Clade F* (red in Figure 1; 93–99 BS and 100 PP) contained previously sampled populations from Oʻahu (F1–2), and new localities from Maui (F3) and Hawaiʻi (F4-F8). We recovered three lineages within *Clade F*: (1) an Oʻahu lineage (F1–2; 63–86 BS and 91–100 PP) that corresponds to one of the *L. hawaiensis* Oʻahu clades reported by Taiti et al. [19]; (2) a new lineage from Hawaiʻi (F4-F8; 81–91 BS and 89–99 PP); and (3) a new lineage formed by a single population from Maui (F3). Maximum COI K2P divergence among *Clade F* lineages was 5.30%.

### Nuclear gene patterns

**Figure 3:**
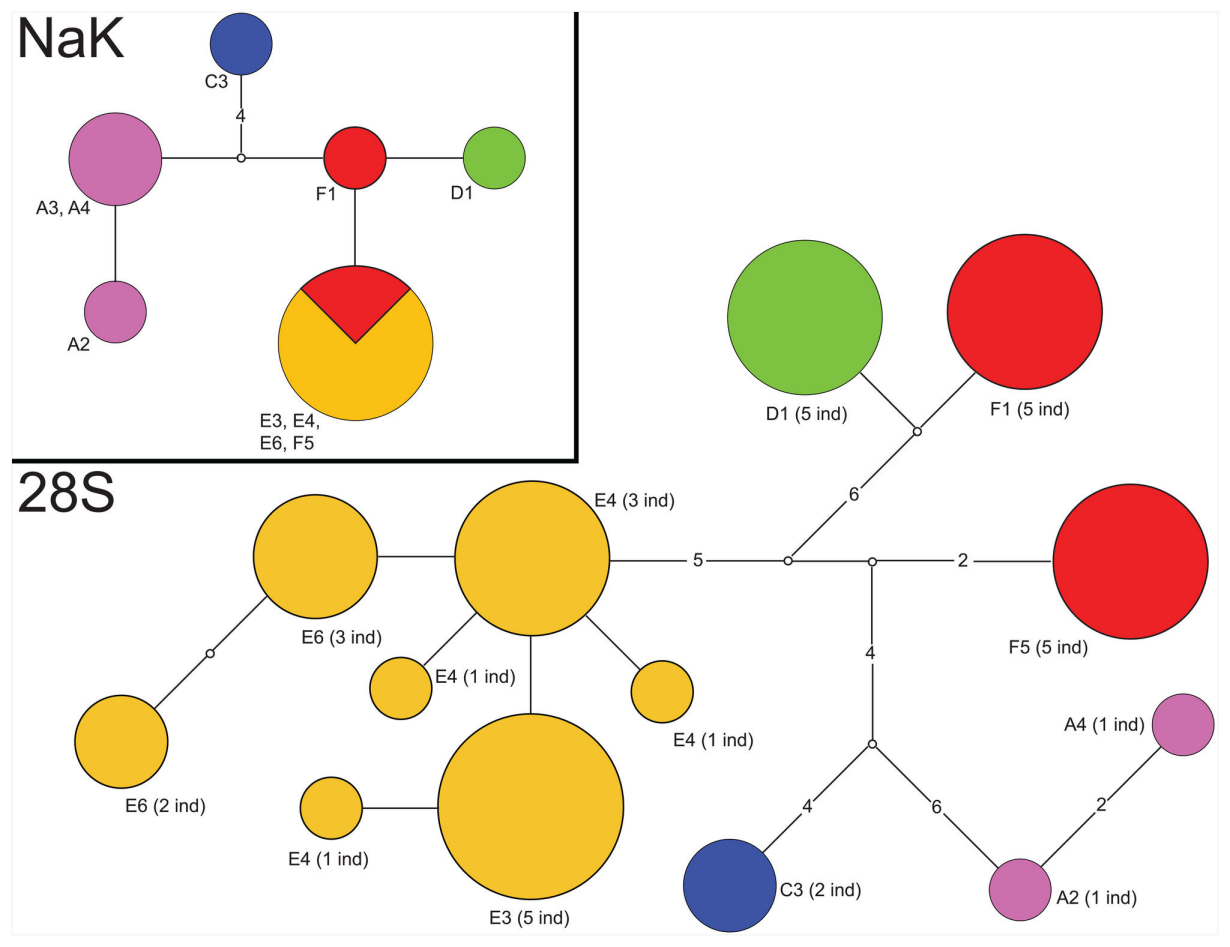
Haplotype networks for two nuclear gene fragments for Ligia from the Hawaiian Archipelago. The nuclear markers examined are the alpha subunit of the Sodium-Potassium ATPase (NaK) and the 28S rDNA gene. Colors correspond with those in Figure 1. Locality ID is indicated next to each allele (locality information is provided in Figure 1 and Table 1). Empty circles represent inferred unsampled (i.e., missing) alleles. Numbers within branches indicate number of mutational steps separating haplotypes. The size of circles is proportional to the frequency at which an allele was recovered. Numbers in parenthesis, indicate the number of individuals observed with the corresponding allele.

We sequenced two nuclear genes for all *Study Area* lineages with the exception of the *L. perkinsi* lineage from Oʻahu (*B*). Multiple attempts to amplify nuclear genes from this population proved unsuccessful. The patterns inferred from the nuclear genes (Figure 3; colors for clades correspond with those in Figure 1) were, in general, consistent with those inferred from the mitochondrial genes. For the NaK gene (Dataset S4), we only observed six alleles, separated by 1–6 steps. For *Clade A* members, we detected two alleles separated by a single step. These alleles were separated from the other alleles by 2–6 steps, which is concordant with the high divergence observed in mitochondrial genes between *Clade A* and all other lineages. The allele observed for the individual of *Clade C* (*L. perkinsi* from Kauaʻi) was divergent from the other alleles by 5–6 steps, also consistent with the mitochondrial results. The NaK results show a closer relationship among members of clades *D*, *E*, and *F*, also congruent with the mitochondrial results. Three alleles were observed for members of these clades, which were separated by only 1–2 steps, with *Clade E* members from Lanaʻi, Molokaʻi and Maui sharing an allele with a *Clade F* member from Hawaiʻi (F5); whereas another member of the *Clade F* from Oʻahu (F1) harbored a unique allele. The *Clade D* individual from Kauaʻi harbored a unique allele.

For the 28S rDNA gene, we excluded 60 poorly aligned positions, resulting in a final alignment of 967 characters, 31 of which were parsimony informative (Dataset S3). For this gene, we detected thirteen alleles. Seven of these alleles were recovered from *Clade E* individuals (Figure 3), with the other lineages harboring one or two alleles. Concordant with mitochondrial phylogenetic findings, alleles from different major lineages appeared highly differentiated, with most lineages separated by 10–21 steps. We note, however, that the single allele found in *Clade D* (Kauaʻi; D1) was separated by only two steps from one of the two alleles observed in *Clade F* (from the Oʻahu F1 locality). The F1 and D1 alleles were in turn separated by 11 steps from the other *Clade F* allele (from the Hawaiʻi F5 locality). This pattern could be the result of incomplete lineage sorting or a past hybridization event. Examination of additional informative nuclear markers in individuals from multiple populations per clade is needed to resolve this question. Indeed, multispecies coalescent analyses of numerous unlinked markers are likely needed to resolve relationships with more certainty [76]. Nevertheless, as shown by Mateos et al. [77], datasets with three loci, including one with strong phylogenetic signal (i.e., the mitochondrial dataset) and one or two with low phylogenetic signal (e.g. NaK) are not well suited for multispecies coalescent approaches, and different methods lead to different results. Therefore, we consider that at the present stage, the mitochondrial phylogeny represents the most plausible hypothesis.

### Geometric morphometrics

We analyzed a total of 84 individuals from six supralittoral localities (D1, E3, E4, E6, F1, and F5), representing three of the major lineages detected for *L. hawaiensis* and all of the Hawaiian Islands sampled (morphometrics datasets and pictures are available in DataDryad doi:10.5061/dryad.5k56c). We did not include individuals from *Clade A* due to limited sampling (i.e., < 3 individuals per population). Principal component analysis generated 24 non-zero eigenvectors and the first 11 accounted for 96.2% of the variance, and thus were included in posterior analyses. The full factorial MANCOVA yielded no significance for the three-way interaction term, thus we removed it and repeated the analysis. This simpler MANCOVA model (Table 2) yielded significant results for the main effects of Lineage, Sex, and Size (p < 0.0001; η_p_^2^ > 0.4). One two-way interaction (sex × lineage) was significant (p = 0.02), but was weak (η_p_^2^ = 0.2).

**Table 2:** Results of multivariate analyses of covariance examining overall body shape in *Ligia hawaiensis*.

	F	d.f._num_	d.f._den_	*p*	η_p_^2^
**Lineage**	**3.660**	**22**	**120**	**<.0001**	**0.4016**
**Sex**	**5.308**	**22**	**120**	**<.0001**	**0.4932**
**Size**	**5.178**	**11**	**60**	**<.0001**	**0.4870**
Lineage * Sex	**1.553**	**44**	**231.5**	**0.0209**	**0.2279**
Sex * Size	0.936	22	120	0.5498	0.1464
Lineage * Size	0.710	22	120	0.8225	0.1151

Significant effects with a η_p_^2^ value >0.2 are indicated in bold.

Quadratic DFAs of MANCOVA residuals achieved an initial correct assignment of individuals to their lineage in 91.7% of cases. After LOOCV, the successful classification rate dropped to 59.5%, with per-lineage validated correct classification rates as follows: 26.7% for *Clade D*, 70.6% for *Clade E*, and 62.9% for *Clade F*. Individuals from *Clade D* were more likely to be identified as *Clade F* (7/15) than to their true lineage (4/15). Most misclassified individuals after LOOCV were assigned to *F* (17 of 34) and *E* (14 of 34) (Table 3). 

**Table 3:** Classification rates for *Ligia hawaiensis* Clade DFAs.

	*Clade D*	*Clade E*	*Clade F*
*Clade D*	**100**	0.00	0.00
	**26.67**	46.67	26.66
*Clade E*	2.63	**89.47**	7.89
	8.57	**62.86**	28.57
*Clade F*	0.00	8.11	**91.89**
	0.00	29.41	**70.59**

Rows indicate actual clade of origin, while columns indicate predicted clade membership. We present the percentage of individuals correctly assigned to their clade of origin for the original model first, followed by LOOCV rates.

### Shape variation in *L. hawaiensis*

We present thin-plate-spline transformations, which represent the average shape deformation, for the three supralittoral lineages examined (*D*, *E*, and *F*; Figure 2B). Along with the visualizations we present photographs of individual specimens having the greatest canonical axis scores. The three lineages examined differ most prominently in two aspects of shape: the width of the body, relative to the total body length, especially in the mid-body, and head size and conformation, particularly with respect to the distance between the eyes, as measured by the distance between their medial boundaries. We also detected minor differences in the distal-most point of the pleotelson. On average, individuals from *Clade D* have an oblong-ovate body with a mid-body narrower than individuals from clades *E* and *F*, and the distance between the eyes appears to be ~2/3 of the total eye width. *Clade E* individuals exhibit, on average, an ovoid shape and eyes that appear to be separated by a distance similar or greater than the total eye width, and the distal-most point of the pleotelson protrudes more prominently than in either *Clade D* or F. Individuals from *Clade F*, on average, exhibit an overall body shape anteriorly broadened relative to those from *Clade D* or *E*; a small head size with less crowding of the eyes than in *Clade D*; and distance between the eyes appears to be equal to the width of the eyes. Finally *Clade F* had a less prominently protruding pleotelson. It should be kept in mind that these morphological differences are those due to clade effects estimated in the overall context of the MANCOVA, while controlling for allometry, sex/maturity status, and clade-specific allometry.

We also present thin-plate-spline transformations for sex/maturity categories (Figure S2 A). Differences between the sexes appear to be mostly localized in the anterior end of the body, with females having wider segments than both males and juveniles. On average, juveniles and males appear to have very similar body shapes, but the latter appear to have less convexity of the body around the midline. Shape differences between sizes were also evident, with larger individuals having a more elongated body and smaller head, relative to smaller individuals (Figure S2 B).

## Discussion

In this study, we conducted phylogeographic analyses of the *Ligia* lineages endemic to the Hawaiian Archipelago including a comprehensive sampling of these isopods across the main Hawaiian Islands, which greatly enhances our understanding on the diversity and evolution of this group. Previous genetic work by Taiti et al. [19] on *Ligia* from the Hawaiian islands of Kauaʻi and Oʻahu reported highly divergent lineages of the terrestrial *L. perkinsi* and the coastal *L. hawaiensis* in these islands. By including populations from previously unsampled main Hawaiian Islands, we detected additional lineages of *L. hawaiensis* and expanded the known range of others. We report the discovery of a new lineage of *L. hawaiensis* (i.e., *Clade A*), distributed in Maui and Hawaiʻi, which is highly divergent (> 10% COI K2P) from previously reported *Ligia* lineages in the Hawaiian Archipelago. We also discovered that the distribution of a lineage (*Clade E*) previously sampled in a locality in Oʻahu (E1) extends to Molokaʻi, Lanaʻi, and Maui. Similarly, we discovered that *Clade F*, which was previously identified in Taiti et al. [19] from Oʻahu, contains two additional lineages; one distributed in Maui (F3) and one in Hawaiʻi (F4-F8). Divergence among the three *Clade F* lineages is > 3% COI K2P. Considering that intra-specific divergences in marine invertebrates are typically < 3% for the same COI fragment used in this study [78], it is likely that some of the divergent Hawaiian *Ligia* lineages represent cryptic species, thus, the taxonomy of the group needs revision.

High levels of genetic differentiation among populations of the Hawaiian *Ligia* are congruent with studies of *Ligia* in other parts of the world ([30-32] and unpublished data). Biological characteristics of these isopods severely restrict their dispersal potential, contributing effectively to the isolation of populations, and, in the long-term, to allopatric genetic differentiation [30]. Nonetheless, phylogeographic patterns indicate that past dispersal events have been important in shaping the evolutionary history of these isopods in the Hawaiian Archipelago.

The monophyly of the endemic Hawaiian *Ligia* lineages is well supported, suggesting that evolution of this group likely occurred within the archipelago. Lineages identified as the closest relatives of the endemic Hawaiian *Ligia* are highly divergent and found in other Pacific localities (unpublished data). Large divergences observed among Hawaiian *Ligia* lineages also suggest a long evolutionary history for this group. In addition, the phylogeographic patterns observed among Hawaiian *Ligia*, although not fully resolved, do not support simpler patterns of evolution, such as colonization from older to younger islands (i.e., progressive rule), or vice versa (i.e., from younger to older islands), but rather a complex evolutionary history. Illustrating the complexity of the evolutionary history of the Hawaiian *Ligia*, the three most basal lineages include one found only in the younger islands (*Clade A*), a terrestrial lineage restricted to Oʻahu (*Clade B*), and one that is the most diverse. This latter clade (*CDEF*) includes supralittoral and terrestrial lineages, and divergent lineages restricted either to the older island of Kauaʻi or to the other islands. Dispersal and local extinctions likely contributed to shape this complex evolutionary history.

Despite the complex evolutionary history of Hawaiian *Ligia*, some phylogeographic patterns emerge that appear congruent with the geological history of the main Hawaiian Islands. First, Kauaʻi, the oldest of the main Hawaiian Islands, harbors only one endemic *L. hawaiensis* lineage (*Clade D*), which is highly divergent from the other lineages. This is consistent with the older geological history of this island and its high degree of isolation, as no overland connections are thought to have existed between Kauaʻi and other Hawaiian islands [37]. Second, in contrast to the pattern observed in Kauaʻi, sharing of highly divergent lineages is observed among the other main Hawaiian Islands, suggesting inter-island dispersal among these islands. The geological history of these islands may have provided opportunities for the exchange of colonizers from divergent lineages. Molokaʻi, Maui, Lanaʻi, and Kahoʻolawe, are thought to have existed as a single landmass, the Maui Nui Complex, throughout most of their geological history, first splitting up some ~0.6 million years ago (Mya) and retaining land connections during glacial low sea level stands [79]. The Maui Nui complex is thought to have been connected via a land bridge to Oʻahu between 2.2–1.9 Mya forming the short lived Oʻahu Nui complex [37,79]. These past connections may have facilitated the dispersal of *Clade E*, restricted to the Maui Nui islands and Oʻahu. The low genetic divergences observed among *Clade E* populations (< 2.5% COI K2P) may indicate a recent history of isolation among populations of this clade. Dispersal of *Clade F* across these islands may also have been facilitated by these connections. Third, historically, the oceanic channel separating Maui and Hawaiʻi does not appear to constitute a very effective barrier for inter-island dispersal of coastal organisms that disperse through rafting, as two divergent lineages are shared between Hawaiʻi and Maui (i.e., clades *A* and *F*). Remarkably, however, members from *Clade E* were not found in Hawaiʻi, despite their wide distribution in the Maui Nui islands.

Evolution of terrestriality appears to have occurred early during the diversification of the Hawaiian *Ligia*, as clades *B* and *C* are highly divergent and occupy a relatively basal position in the phylogeny of Hawaiian *Ligia*. Our results confirm the paraphyly of *L. perkinsi*, previously observed by Taiti et al. [19]. Therefore, from the phylogeographic patterns it is uncertain (1) whether evolution of terrestriality occurred independently in each island (i.e., Kauaʻi and Oʻahu) or (2) whether terrestriality evolved once. Both of the above hypotheses are equally parsimonious assuming a coastal ancestor for the endemic Hawaiian *Ligia* clade, which we consider most likely, as the closest lineages are coastal and because of the predominantly coastal nature of the genus. In addition, the remoteness of the Hawaiian archipelago makes colonization by a coastal lineage more likely than by a terrestrial one. Under the observed phylogenetic patterns, an independent origin of terrestriality would have required two steps: the evolution of terrestriality along the branches of clades *B* and *C*. Conversely, a single origin of terrestriality may only be explained by invoking the adaptation to terrestrial habitats followed by a reversal to the marine habitat (i.e., 2 steps). Taiti et al. [19] considers the independent adaptation to terrestrial habitats in Hawaiian *Ligia* as the most plausible explanation for the origin of *L. perkinsi* populations in Oʻahu and Kauaʻi. They consider a shift from terrestrial to seashore conditions unlikely, given that most of the species in the genus occupy supralittoral habitats, and that terrestrial forms appear to have derived from supralittoral forms [28]. Similar conditions could have acted in Oʻahu and Kauaʻi that facilitated the independent colonization of freshwater habitats. Nonetheless, not enough is known about the biology of *L. perkinsi* to discard the possibility of a reversal. This species is often found in the rocky shores of streams [34], with populations in Oʻahu occurring less than 3 Km from shore and at low altitude [80]. Furthermore, *L. perkinsi* exhibits the highest osmoregulatory capabilities observed among *Ligia* species [81]. Post-hoc analyses from Approximately Unbiased (AU) tests implemented in CONSEL [82] rejected the monophyly of *L. perkinsi* (p = 1 × 10^-6^), which would have supported a single origin of terrestriality. These tests also rejected the monophyly of *L. hawaiensis* (p = 2 × 10^-5^), as well as the monophyly of *L. perkinsi* from Kauaʻi + *L. hawaiensis* from Kauaʻi (p = 1 × 10^-5^).

Similar to the Hawaiian *Ligia*, the shrimp *Halocaridinia rubra*, which inhabits anchialine coastal pools, also shows multiple highly divergent lineages, with allopatric distributions [17,18]. In contrast with *Ligia*, however, presence of a highly divergent lineage in multiple islands is rare in the shrimp, with only one case reported among the three islands studied (i.e., Oʻahu, Maui, and Hawaiʻi). A shrimp lineage that was found mainly on the west coast of Hawaiʻi was also found in one locality of Oʻahu, represented by a single haplotype that was very close (only one substitution) to some haplotypes present in Hawaiʻi. Therefore, inter-island dispersal appears to have been more limited in this shrimp.

Morphological differences have been previously reported between *L. perkinsi* lineages (B, C), but not between *L. hawaiensis* lineages (D, F) [19]. Previous comparisons, however, relied on classic taxonomic characters. Geometric-morphometric approaches have proven useful in identifying differences between otherwise cryptic species in other invertebrate taxa [83-86], including crustaceans [87,88]. They also have been used to test body shape differences at the intraspecific level in isopods [89]. Our geometric-morphometric analyses revealed significant differences in body shape among the three highly genetically divergent supralittoral lineages examined (*D*, *E*, and *F*). Rates of correct assignment of quadratic DFAs after LOOCV were low, however, indicating body shape alone is of limited use as a taxonomic diagnostic tool. On average, lineages differ in traits widely used in the taxonomy of *Ligia*, including relative body width, distance between the eyes, and the protrusion of the distal-most point of the pleotelson [19,25,27,66,67]. Interestingly, individuals from *Clade D* exhibit average differences with respect to the other two clades in traits that were previously used to describe *Ligia kauaiensis*, such as a narrow oblong-ovoid body shape and shorter distances between the eyes [90]. This species, now considered a synonym of *L. hawaiensis* [20,91], was first described from individuals from the shores of Kalihiwai Bay, Kauaʻi [90], the same location (D1) included in our molecular and morphometric analyses. Therefore, the deep genetic divergence of *Clade D* (at least 13.57% COI K2P), its discrete geographic distribution, and apparently distinct morphology suggest that *L. kauaiensis* may be a valid species. 

We also detected significant differences in the average overall body shape between sexes, with females exhibiting wider anterior segments than males and juveniles. These differences may be caused by the development of the ventral marsupium (i.e., brood pouch) in females. This structure forms from thoracic sterna and overlapping oostegites prior to copulation in mature females, and was present in all samples identified as females. As we did not observe obvious differences between males and juveniles, and females were classified based on the presence of the marsupium, a temporary structure [92], differences between the sexes in *L. hawaiensis* may be temporary, and thus, not relevant to the taxonomy of *Ligia* in the Hawaiian Archipelago. Lastly, we detected an effect of body size on body shape for *L. hawaiensis*, with larger individuals exhibiting a more elongated body with relatively smaller head than smaller individuals. Allometry has been reported also for *Ligia pallasii*, where in contrast to our results, larger individuals (usually males) tend to be disproportionality broader than smaller ones [93,94], a pattern that we have also observed in other *Ligia* from the northern East Pacific (unpublished data).

## Conclusions

Our study revealed new lineages and expanded the distribution range of several lineages of supralittoral *Ligia* in the Hawaiian Archipelago. A previously unknown highly divergent lineage (*Clade A*) of supralittoral *Ligia* was found in the islands of Hawaiʻi and Maui. We found that *Clade E*, a lineage previously reported only from Oʻahu, is widely distributed in the Maui-Nui islands. *Clade F*, a lineage also previously reported only from Oʻahu, contains two additional moderately divergent lineages with geographically restricted distributions; one in Maui and one in Hawaiʻi. Our results supported the monophyly of all *Ligia* lineages endemic to the Hawaiian Archipelago, consistent with a single colonization event. Our results, however, did not support the monophyly of all the supralittoral lineages (currently grouped into *L. hawaiensis*), and/or the monophyly of the terrestrial lineages (currently grouped into *L. perkinsi*), implying more than one evolutionary transition between coastal and inland forms. The lack of monophyly of currently recognized species and the relatively high genetic divergences of several lineages argues for a taxonomic revision. The phylogeographic patterns of *Ligia* in the study area are complex, inconsistent with simple evolutionary patterns proposed for the Hawaiian Islands, such as the progression rule. Evidence for multiple historical dispersal events between islands is observed among Hawaiʻi, Oʻahu, and the Maui-Nui islands. In contrast, the oldest and most geographically distant of the major islands (Kauaʻi) shares no lineages with the other islands. Although multiple lineages inhabit several islands, no instances of sympatry at the locality level were detected. Significant body shape differences were observed among three *L. hawaiensis* clades examined (*D*, *E*, and *F*), one of which was consistent with previous recognition of a separate coastal species in Kauaʻi (i.e., *L. kauaiensis*).

Finally, our results are relevant for the protection of unique and highly localized biodiversity found in an environment subject to high pressure from human disturbances in the Hawaiian Islands. Destruction and modification of rocky intertidal beaches through activities such as construction and conversion to sandy beaches (e.g. by sand nourishing) can destroy the natural habitat of *Ligia* and, thus, jeopardize the survival of unique lineages endemic to the Hawaiian Islands.

## Supporting Information

Dataset S1 **Alignment of COI gene sequences for all sequenced individuals and *L. hawaiensis* sequences from GenBank.**
(NEX)

Dataset S2 **Alignment of concatenated mitochondrial genes dataset; positions excluded from phylogenetics analyses are indicated.**
(NEX)

Dataset S3 **Alignment of the 28S rDNA sequences; positions excluded from analyses are indicated.**
(NEX)

Dataset S4 **Alignment of NaK gene sequences.**
(NEX)

Figure S1 **Neighbor-Joining tree of COI haplotypes (Dataset S1), including 1–10 individuals per locality. Colored taxon labels represent those used in concatenated mitochondrial phylogenetic analyses.** Tree is available at TreeBase (http://purl.org/phylo/treebase/phylows/study/TB2:S14886). * denotes individuals for which the 28S rDNA gene was successfully sequenced, whereas ° denotes those for which the NaK gene was sequenced.
(PDF)

Figure S2 **Shape effect canonical axes for *Ligia hawaiensis* morphology based on sex and body size.** A. Multivariate centroid and 95% confidence ellipses for females (F), juveniles (J) and males (M). Axes are scaled to reflect the magnitude of each relative to the other. B. Individual canonical scores for the size effect on shape. Visualizations were made using canonical scores in the software program tpsRegr v1.49 and are scaled to reflect approximately the range of variation observed in our samples.
(PDF)

Table S1 **Localities included in the study, with corresponding GenBank accession numbers, and geographic information.**
(DOCX)

Table S2 **Included and excluded characters and substitution models used for phylogenetic reconstructions.**
(DOCX)

Table S3 **Settings for phylogenetic analyses of the concatenated mitochondrial (MT) dataset.**
(DOCX)

Table S4 **Support values (percentage) for phylogenetic analyses of the concatenated mitochondrial (MT) dataset.**
(DOCX)

## References

[1] Wagner WL, Funk VA (1995) Hawaiian Biogeography: Evolution on a Hot Spot Archipelago Washington, D.C. (USA). Smithsonian Institution Press.

[2] Carson HL (1982) Evolution of *Drosophila* on the newer Hawaiian volcanoes. Heredity (Edinb) 48: 3-25. doi:10.1038/hdy.1982.2. PubMed: 7042651. 7042651

[3] O'Grady P, DeSalle R (2008) Out of Hawaii: the origin and biogeography of the genus *Scaptomyza* (Diptera: Drosophilidae). Biol Lett 4: 195-199. doi:10.1098/rsbl.2007.0575. PubMed: 18296276. 18296276 PMC2429922

[4] Carson HL, Kaneshiro KY (1976) *Drosophila* of Hawaii: systematics and ecological genetics. Annu Rev Ecol Syst 7: 311-345. doi:10.2307/2096870.

[5] Baldwin BG, Robichaux RH (1995) Historical biogeography and ecology of the Hawaiian silversword alliance (Asteraceae). In: Wagner WL Funk VA Hawaiian Biogeography: Evolution On A Hot Spot Archipelago. Washington D.C.: Smithsonian Institution Press pp. 259–287.

[6] Rundell RJ, Holland BS, Cowie RH (2004) Molecular phylogeny and biogeography of the endemic Hawaiian Succineidae (Gastropoda: Pulmonata). Mol Phylogenet Evol 31: 246-255. doi:10.1016/j.ympev.2003.07.014. PubMed: 15019623. 15019623

[7] Holland BS, Hadfield MG (2004) Origin and diversification of the endemic Hawaiian tree snails (Achatinellidae: Achatinellinae) based on molecular evidence. Mol Phylogenet Evol 32: 588-600. doi:10.1016/j.ympev.2004.01.003. PubMed: 15223040. 15223040

[8] Fleischer RC, McIntosh CE, Tarr CL (1998) Evolution on a volcanic conveyor belt: using phylogeographic reconstructions and K–Ar-based ages of the Hawaiian Islands to estimate molecular evolutionary rates. Mol Ecol 7: 533-545. doi:10.1046/j.1365-294x.1998.00364.x. PubMed: 9628004. 9628004

[9] Tarr CL, Fleischer RC (1995) Evolutionary relationships of the Hawaiian honeycreepers (Aves, Drepanidinae). In: Wagner WL Funk VA Hawaiian Biogeography: Evolution on a Hot Spot Archipelago. Washington, D.C.: Smithsonian Institution Press pp. 147–159.

[10] Gillespie RG, Croom HB, Palumbi SR (1994) Multiple origins of a spider radiation in Hawaii. Proc Natl Acad Sci U S A 91: 2290-2294. doi:10.1073/pnas.91.6.2290. PubMed: 8134390. 8134390 PMC43356

[11] Jordan S, Simon C, Foote D, Englund RA (2005) Phylogeographic patterns of Hawaiian *Megalagrion* damselflies (Odonata: Coenagrionidae) correlate with Pleistocene island boundaries. Mol Ecol 14: 3457-3470. doi:10.1111/j.1365-294X.2005.02669.x. PubMed: 16156815. 16156815

[12] Magnacca KN, Danforth BN (2006) Evolution and biogeography of native Hawaiian *Hylaeus* bees (Hymenoptera: Colletidae). Cladistics 22: 393-411. doi:10.1111/j.1096-0031.2006.00119.x.

[13] Rubinoff D (2008) Phylogeography and ecology of an endemic radiation of Hawaiian aquatic case-bearing moths (*Hyposmocoma*: Cosmopterigidae). Philos Trans R Soc Lond B Biol Sci 363: 3459-3465. doi:10.1098/rstb.2008.0115. PubMed: 18765359. 18765359 PMC2607376

[14] Kay AE, Palumbi SR (1987) Endemism and evolution in Hawaiian marine invertebrates. Trends Ecol Evol 2: 183-186. doi:10.1016/0169-5347(87)90017-6. PubMed: 21227847. 21227847

[15] Bird CE, Holland BS, Bowen BW, Toonen RJ (2011) Diversification of sympatric broadcast-spawning limpets (*Cellana* spp.) within the Hawaiian archipelago. Mol Ecol 20: 2128-2141. doi:10.1111/j.1365-294X.2011.05081.x. PubMed: 21481050. 21481050

[16] Hurtado LA, Lee EJ, Mateos M (2013) Contrasting phylogeography of sandy vs. rocky supralittoral isopods in the megadiverse and geologically dynamic Gulf of California and adjacent areas. PLOS ONE 8: e67827. doi:10.1371/journal.pone.0067827. PubMed: 23844103. 23844103 PMC3699670

[17] Craft JD, Russ AD, Yamamot MN, Iwai TY, Hau S et al. (2008) Islands under islands: The phylogeography and evolution of *Halocaridina* *rubra* Holthuis, 1963 (Crustacean: Decapoda: Atyidae) in the Hawaiian archipelago. Limnol Oceanogr 53: 675-689. doi:10.4319/lo.2008.53.2.0675.

[18] Santos SR (2006) Patterns of genetic connectivity among anchialine habitats: a case study of the endemic Hawaiian shrimp *Halocaridina* *rubra* on the island of Hawaii. Mol Ecol 15: 2699-2718. doi:10.1111/j.1365-294X.2006.02965.x. PubMed: 16911195. 16911195

[19] Taiti S, Arnedo MA, Lew SE, Roderick GK (2003) Evolution of terrestriality in Hawaiian species of the genus *Ligia* (Isopoda, Oniscidea). Crustaceana Monographs 2: 85-102.

[20] Schmalfuss H (2003) World catalog of terrestrial isopods (Isopoda: Oniscidea). Stuttgarter Beitr Naturkd Ser A 654: 1-341.

[21] Taiti S, Ferrara F (2004) The terrestrial Isopoda (Crustacea: Oniscidea) of the Socotra Archipelago. Fauna of Arabia 20: 211-326.

[22] Nunomura N, Horiguchi H, Sasaki T, Hironaka M, Hariyama T (2011) A new species of the genus *Ligia* (Crustacea: Isopoda: Ligiidae) from steep streams of Chichijima and Anijima Islands of the Ogasawara Islands. Bulletin of the Toyama Science Museum 34: 73-79

[23] Nunomura N (2009) Terrestrial isopod crustaceans from Daito Islands, southern Japan. Bulletin of the Toyama Science Museum 32: 75-87

[24] Nunomura N (2001) Terrestrial isopod crustaceans from Saipan, Northern Mariana, Micronesia. Bulletin of the Toyama Science Museum 24: 1-17

[25] Khalaji-Pirbalouty V, Wägele JW (2010) Two new species of Ligia Fabricius, 1798 (Crustacea: Isopoda: Ligiidae) from coasts of the Persian and Aden gulfs. Org Divers Evol 10: 135-145. doi:10.1007/s13127-010-0003-5.

[26] Tsuge M (2008) A new species of the genus *Ligia* (Crustacea: Isopoda: Ligiidae) from the Lake Shinji (Shimane Prefecture), western Japan. Bulletin of the Toyama Science Museum 31: 51

[27] Jackson HG (1922) A revision of the isopod genus *Ligia* (Fabricius). Proc Zool Soc Lond 92: 683-703. doi:10.1111/j.1096-3642.1922.tb02164.x.

[28] Schmalfuss H (1979) *Ligia* *simoni*. A model for the evolution of terrestrial isopods. Staatliches Museum für Naturkunde in Stuttgart 317: 1-5.

[29] Carefoot TH, Taylor BE (1995) *Ligia*: a prototypal terrestrial isopod. In: Alikhan MA Terrestrial isopod biology. Rotterdam: A.A. Balkema Publishers pp. 47-60.

[30] Hurtado LA, Mateos M, Santamaria CA (2010) Phylogeography of supralittoral rocky intertidal *Ligia* isopods in the Pacific region from Central California to Central Mexico. PLOS ONE 5: e11633. doi:10.1371/journal.pone.0011633. PubMed: 20657776. 20657776 PMC2908127

[31] Eberl R, Mateos M, Grosberg RK, Santamaria CA, Hurtado LA (2013) Phylogeography of the supralittoral isopod *Ligia* *occidentalis* around the Point Conception marine biogeographical boundary. J Biogeogr 40: 2361–2372. doi:10.1111/jbi.12168.

[32] Jung J, Eo HS, Rho RS, Kim W (2008) Two genetic lineages of sea slaters, *Ligia* (Crustacea: Isopoda) in South Korea: a population genetic approach. Mol Cells 25: 523-530. PubMed: 18443407. 18443407

[33] Vandel A (1960) Isopodes terrestres ( Première partie). Faune France 64: 1-416

[34] Taiti S, Howarth FG (1996) Terrestrial isopods from the Hawaiian Islands (Isopoda: Oniscidea). Bishop Mus Occas Pap 45: 59-71

[35] Eldredge LG, Smith CM, editors (2001) A guidebook of introduced marine species in Hawaii Bishop Museum Technical Report 21 p. 60.

[36] Taiti S, Ferrara F (1991) Terrestrial isopods (Crustacea) from the Hawaiian Islands. Bishop Mus Occas Pap 31: 202-227

[37] Carson HL, Clague DA (1995) Geology and biogeography of the Hawaiian Islands. In: Wagner WL Funk VA Hawaiian Biogeography: Evolution On A Hot Spot Archipelago. Washington, D.C.: Smithsonian Institution Press pp. 14-29.

[38] Folmer O, Black M, Hoeh W, Lutz R, Vrijenhoek R (1994) DNA primers for amplification of mitochondrial cytochrome c oxidase subunit I from diverse metazoan invertebrates. Mol Mar Biol Biotechnol 3: 294-299. PubMed: 7881515. 7881515

[39] Palumbi S (1996) Nucleic Acids II: Polymerase Chain Reaction. In: Hillis D Moritz C Mable B Molecular Systematics. 2nd ed. Sunderland, MA: Sinauer Associates Inc. pp. 205–247.

[40] Podsiadlowski L, Bartolomaeus T (2005) Organization of the mitochondrial genome of mantis shrimp *Pseudosquilla* *ciliata* (Crustacea: Stomatopoda). Mar Biotechnol (NY) 7: 618-624. doi:10.1007/s10126-005-0017-8. PubMed: 16088353. 16088353

[41] Merritt TJS, Shi L, Chase MC, Rex MA, Etter RJ et al. (1998) Universal cytochrome *b* primers facilitate intraspecific studies in molluscan taxa. Mol Mar Biol Biotechnol 7: 7-11. PubMed: 9597773. 9597773

[42] Whiting MF (2002) Mecoptera is paraphyletic: multiple genes and phylogeny of Mecoptera and Siphonaptera. Zool Scripta 31: 93-104. doi:10.1046/j.0300-3256.2001.00095.x.

[43] Tsang LM, Ma KY, Ahyong ST, Chan TY, Chu KH (2008) Phylogeny of Decapoda using two nuclear protein-coding genes: origin and evolution of the Reptantia. Mol Phylogenet Evol 48: 359-368. doi:10.1016/j.ympev.2008.04.009. PubMed: 18501643. 18501643

[44] Katoh K, Kuma K, Toh H, Miyata T (2005) MAFFT version 5: improvement in accuracy of multiple sequence alignment. Nucleic Acids Res 33: 511-518. doi:10.1093/nar/gki198. PubMed: 15661851. 15661851 PMC548345

[45] Penn O, Privman E, Ashkenazy H, Landan G, Graur D, et al. (2010) GUIDANCE: a web server for assessing alignment confidence scores. Nucleic Acids Res 38 (Web Server issue): W23-W28 doi: 10.1093/nar/gkq443. PMC2896199 20497997

[46] Penn O, Privman E, Landan G, Graur D, Pupko T (2010) An alignment confidence score capturing robustness to guide tree uncertainty. Mol Biol Evol 27: 1759-1767. doi:10.1093/molbev/msq066. PubMed: 20207713. 20207713 PMC2908709

[47] Tamura K, Peterson D, Peterson N, Stecher G, Nei M et al. (2011) MEGA5: Molecular evolutionary genetics analysis using maximum likelihood, evolutionary distance, and maximum parsimony methods. Mol Biol Evol 28: 2731-2739. doi:10.1093/molbev/msr121. PubMed: 21546353. 21546353 PMC3203626

[48] Darriba D, Taboada GL, Doallo R, Posada D (2012) jModelTest 2: more models, new heuristics and parallel computing. Nat Methods 9: 772-772. doi:10.1038/nmeth.2109. PMC4594756 22847109

[49] Yang Z (2006) Computational Molecular Evolution; Harvey PH May RM New York, NY: Oxford University Press p. 357.

[50] Lanfear R, Calcott B, Ho SYW, Guindon S (2012) PartitionFinder: combined selection of partitioning schemes and substitution models for phylogenetic analyses. Mol Biol Evol 29: 1695-1701. doi:10.1093/molbev/mss020. PubMed: 22319168. 22319168

[51] Stamatakis A (2006) RAxML-VI-HPC: maximum likelihood-based phylogenetic analyses with thousands of taxa and mixed models. Bioinformatics 22: 2688-2690. doi:10.1093/bioinformatics/btl446. PubMed: 16928733. 16928733

[52] Stamatakis A, Hoover P, Rougemont J (2008) A rapid bootstrap algorithm for the RAxML web servers. Syst Biol 57: 758-771. doi:10.1080/10635150802429642. PubMed: 18853362. 18853362

[53] Stamatakis A (2006) Phylogenetic models of rate heterogeneity: a high performance computing perspective. Proceedings of 20th IEEE/ACM International Parallel and Distributed Processing Symposium, Rhodos (Greece). pp. 278-278.

[54] Zwickl DJ (2006) Genetic algorithm approaches for the phylogenetic analysis of large biological sequence datasets under the maximum likelihood criterion. [Ph.D. dissertation]; Austin TX The University of Texas at. 115 p.

[55] Sukumaran J, Holder MT (2010) DendroPy: a Python library for phylogenetic computing. Bioinformatics 26: 1569-1571. doi:10.1093/bioinformatics/btq228. PubMed: 20421198. 20421198

[56] Huelsenbeck JP, Ronquist F (2001) MRBAYES: Bayesian inference of phylogenetic trees. Bioinformatics 17: 754-755. doi:10.1093/bioinformatics/17.8.754. PubMed: 11524383. 11524383

[57] Ronquist F, Huelsenbeck JP (2003) MrBayes 3: Bayesian phylogenetic inference under mixed models. Bioinformatics 19: 1572-1574. doi:10.1093/bioinformatics/btg180. PubMed: 12912839. 12912839

[58] Lewis PO, Holder MT, Swofford DL (2008) Phycas: software for phylogenetic analysis. Available: http://www.phycas.org. 10.1093/sysbio/syu132 25577605

[59] Lewis PO, Holder MT, Holsinger KE (2005) Polytomies and Bayesian phylogenetic inference. Syst Biol 54: 241-253. doi:10.1080/10635150590924208. PubMed: 16012095. 16012095

[60] Suzuki Y, Glazko GV, Nei M (2002) Overcredibility of molecular phylogenies obtained by Bayesian phylogenetics. Proc Natl Acad Sci U S A 99: 16138-16143. doi:10.1073/pnas.212646199. PubMed: 12451182. 12451182 PMC138578

[61] Nylander JAA, Wilgenbusch JC, Warren DL, Swofford DL (2008) AWTY (are we there yet? ): a system for graphical exploration of MCMC convergence in Bayesian phylogenetics. Bioinformatics 24: 581-583 doi: 10.1093/bioinformatics/btm388. 17766271

[62] Rambaut A, Drummond AJ (2009) Tracer v1.5. Available: http://beast.bio.ed.ac.uk/Tracer.

[63] Templeton AR, Crandall KA, Sing CF (1992) A cladistic analysis of phenotypic associations with haplotypes inferred from restriction endonuclease mapping and DNA sequence data. III. Cladogram estimation. Genetics 132: 619-633. PubMed: 1385266. 1385266 10.1093/genetics/132.2.619 PMC1205162

[64] Clement M, Posada D, Crandall A (2000) TCS: a computer program to estimate gene genealogies. Mol Ecol 9: 1657-1659. doi:10.1046/j.1365-294x.2000.01020.x. PubMed: 11050560. 11050560

[65] Rohlf FJ (2004) TpsDig, version 1.40. Available: http://life.bio.sunysb.edu/morph/morphmet/tpsdigw32.exe.

[66] Lee JD (1994) A new mountain slater, *Ligia* *taiwanensis* (Isopoda, Ligiidae) from Taiwan. Crustaceana 66: 110-115. doi:10.1163/156854094X00198.

[67] Schultz GA, Johnson C (1984) Terrestrial isopod crustaceans from Florida (Oniscoidea). Tylidae, Ligiidae, Halophilosciidae, Philosciidae, and Rhyscotidae. J Crustacean Biol 4: 154-171. doi:10.2307/1547904.

[68] Schultz GA (1974) Terrestrial isopod crustaceans mainly from the West Indies and adjacent regions, 1. *Tylos* and *Ligia* . Studies Fauna of Curaçao and Other Caribbean Islands 45: 162-173.

[69] Zelditch ML, Swiderski DL, Sheets HD, Fink WL (2004) Geometric Morphometrics for Biologists: A Primer. New York, NY and London: Elsevier Academic Press.

[70] Rohlf FJ (2006). TpsRelw 1: 44 Available: http://life.bio.sunysb.edu/morph/morphmet/tpsrelww32.exe.

[71] Bookstein FL (1991) Morphometric Tools for Landmark Data. Geometry and Biology. New York, NY: Cambridge University Press.

[72] Tabachnick BG, Fidell LS (2001) Using Multivariate Statistics. Upper Saddle River, NJ. Pearson Allyn & Bacon.

[73] Langerhans RB, DeWitt TJ (2004) Shared and unique features of evolutionary diversification. Am Nat 164: 335-349. doi:10.1086/422857. PubMed: 15478089. 15478089

[74] Rohlf FJ (2005) TpsRegr, version 1.31. Available: http://life.bio.sunysb.edu/morph/morphmet/tpsregrw32.exe.

[75] Smith HL, Smallwood AM, DeWitt TJ (In press) Defining the normative range of Clovis fluted point shape using geographic models of geometric morphometric variation. In: Smallwood AM Jennings TJ Clovis: On the Edge of a New Understanding. College Station, TX: Texas A&M University Press.

[76] Degnan JH, Rosenberg NA (2009) Gene tree discordance, phylogenetic inference and the multispecies coalescent. Trends Ecol Evol 24: 332-340. doi:10.1016/j.tree.2009.01.009. PubMed: 19307040. 19307040

[77] Mateos M, Hurtado LA, Santamaria CA, Leignel V, Guinot D (2012) Molecular systematics of the deep-sea hydrothermal vent endemic brachyuran family Bythograeidae: a comparison of three Bayesian species tree methods. PLOS ONE 7: e32066. doi:10.1371/journal.pone.0032066. PubMed: 22403623. 22403623 PMC3293879

[78] Bucklin A, Steinke D, Blanco-Bercial L (2011) DNA barcoding of marine metazoa. Annu. Rev Mar Sci 3: 471-508. doi:10.1146/annurev-marine-120308-080950Available: . doi:10.1146/annurev-marine-120308-080950. 21329214

[79] Price JP, Elliott-Fisk D (2004) Topographic history of the Maui Nui complex, Hawai'i, and its implications for biogeography. Pac Sci 58: 27-45

[80] Lichtwardt RW (1986) The Trichomycetes. Fungal Associates Of Arthropods. New York, NY: Springer-Verlag.

[81] Carefoot TH, Wright J, Pennings SC, Ziegler A, Zimmer M et al. (2000) Hemolymph homeostasis in relation to diel feeding activity and microclimate in the prototypal land isopod *Ligia* *pallasii* . Can J Zool 78: 588-595. doi:10.1139/z99-234.

[82] Shimodaira H, Hasegawa M (2001) CONSEL: for assessing the confidence of phylogenetic tree selection. Bioinformatics 17: 1246-1247. doi:10.1093/bioinformatics/17.12.1246. PubMed: 11751242. 11751242

[83] Mitrovski-Bogdanovic A, Petrovic A, Mitrovic M, Ivanovic A, Zikic V et al. (2013) Identification of two cryptic species within the *Praon* *abjectum* group (Hymenoptera: Braconidae: Aphidiinae) using molecular markers and geometric morphometrics. Ann Entomol Soc Am 106: 170-180. doi:10.1603/an12100.

[84] Francuski L, Ludoski J, Vujić A, Milankov V (2009) Wing geometric morphometric inferences on species delimitation and intraspecific divergent units in the *Merodon* *ruficornis* group (Diptera, Syrphidae) from the Balkan Peninsula. Zoolog Sci 26: 301-308. doi:10.2108/zsj.26.301. PubMed: 19798925. 19798925

[85] Milankov V, Ludoski J, Stahls G, Stamenkovic J, Vujic A (2009) High molecular and phenotypic diversity in the *Merodon* *avidus* complex (Diptera, Syrphidae): cryptic speciation in a diverse insect taxon. Zool J Linn Soc 155: 819-833. doi:10.1111/j.1096-3642.2008.00462.x.

[86] Carvajal-Rodríguez A, Guerra-Varela J, Fernández B, Rolán E, Rolán-Álvarez E (2006) An example of the application of geometric morphometric tools to the morphological diagnosis of two sibling species in *Nassarius* (Mollusca, Prosobranchia). Iberus 24: 81-88.

[87] Zuykova E, Bochkarev N, Katokhin A (2012) Identification of the *Daphnia* species (Crustacea: Cladocera) in the lakes of the Ob and Yenisei River basins: morphological and molecular phylogenetic approaches. Hydrobiologia 715: 1-16. doi:10.1007/s10750-012-1423-3.

[88] Bertocchi S, Brusconi S, Gherardi F, Buccianti A, Scalici M (2008) Morphometrical characterization of the *Austropotamobius* *pallipes* species complex. J Nat Hist 42: 2063-2077. doi:10.1080/00222930802254664.

[89] Eroukhmanoff F, Svensson EI (2009) Contemporary parallel diversification, antipredator adaptations and phenotypic integration in an aquatic isopod. PLOS ONE 4: e6173. doi:10.1371/journal.pone.0006173. PubMed: 19587791. 19587791 PMC2704376

[90] Edmondson CH (1931) New crustaceans from Kauai, Oahu, and Maui. Occas Pap Bernice P Bishop. Museon 9: 3-18.

[91] Arcangeli A (1954) *Ligyda* *kauaiensis* Edmonson 1931 e *Ligia* *callani* Collinge 1946 sono sinonimi di specie già note (Crostacei Isopodi terrestri). Atti dell’Accademia di Scienze di Torino 88: 147-152

[92] Hornung E (2011) Evolutionary adaptation of oniscidean isopods to terrestrial life: structure, physiology and behavior. Terr Arthropod Rev 4: 95-130. doi:10.1163/187498311X576262.

[93] Eberl R (2012) Distribution, habitat and food preferences of sympatric high intertidal isopod species *Ligia* *occidentalis* and *Ligia* *pallasii* (Ligiidae: Oniscidea). J Nat Hist 46: 1779-1797. doi:10.1080/00222933.2012.700334.

[94] Carefoot TH (1973) Studies on growth, reproduction, and life-cycle of supralittoral isopod *Ligia* *pallasii* . Mar Biol 18: 302-311.

