# Supplementary figures and images for: A Complex Evolutionary History in a Remote Archipelago: Phylogeography and Morphometrics of the Hawaiian Endemic Ligia Isopods
Source: PLoS One. 2013 Dec 30;8(12):e85199. doi: 10.1371/journal.pone.0085199 (PMC3875554; doi:10.1371/journal.pone.0085199)

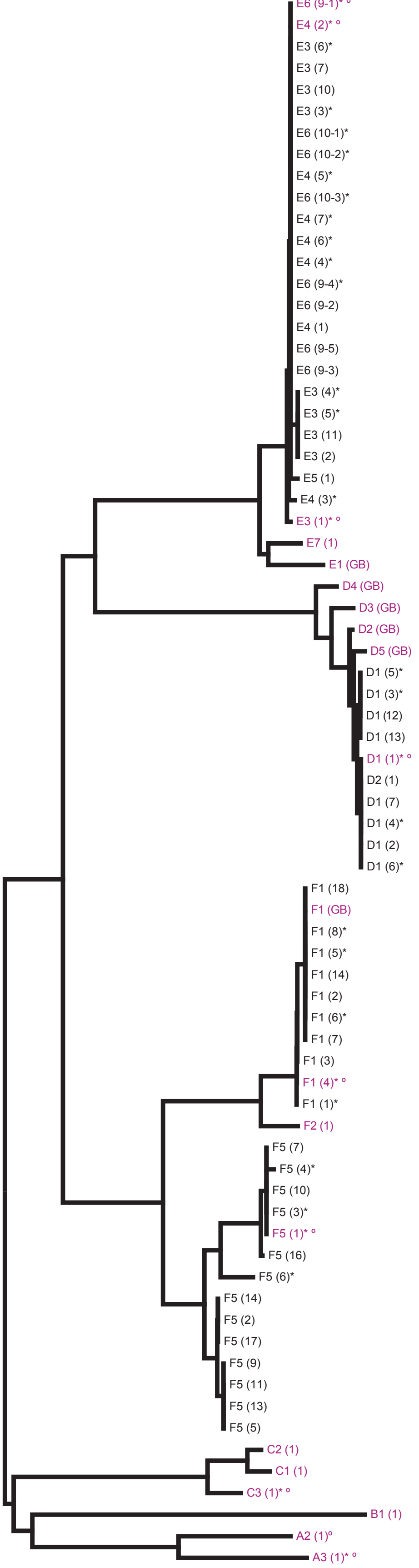

0.01

Supplement: Figure S1 — Neighbor-Joining tree of COI haplotypes (Dataset S1), including 1–10 individuals per locality. Colored taxon labels represent those used in concatenated mitochondrial phylogenetic analyses. Tree is available at TreeBase (http://purl.org/phylo/treebase/phylows/study/TB2:S14886). * denotes individuals for which the 28S rDNA gene was successfully sequenced, whereas ° denotes those for which the NaK gene was sequenced. (PDF) [file pone.0085199.s005.pdf]

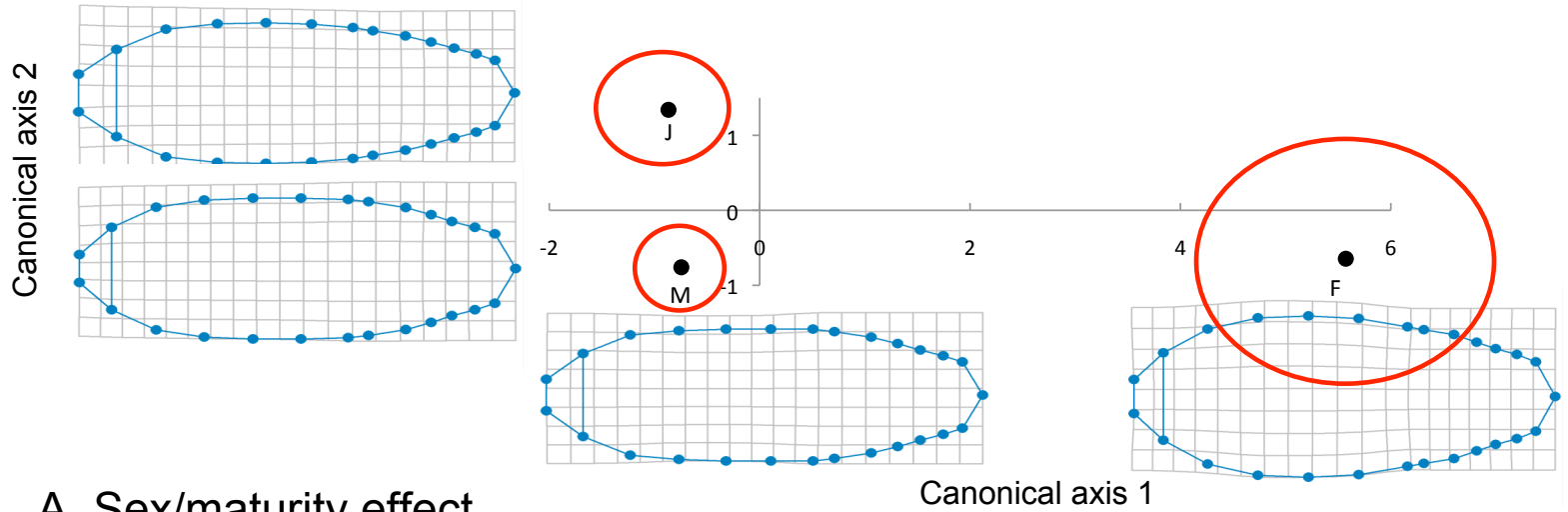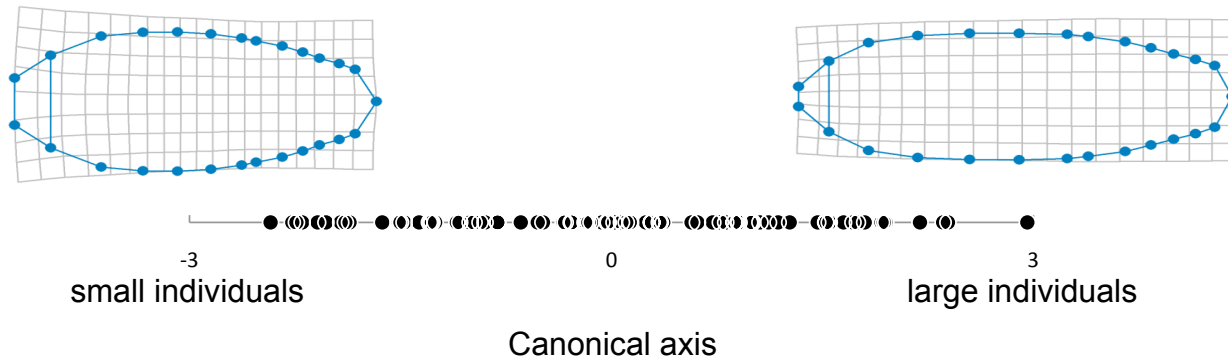

Supplement: Figure S2 — Shape effect canonical axes for Ligia hawaiensis morphology based on sex and body size. A. Multivariate centroid and 95% confidence ellipses for females (F), juveniles (J) and males (M). Axes are scaled to reflect the magnitude of each relative to the other. B. Individual canonical scores for the size effect on shape. Visualizations were made using canonical scores in the software program tpsRegr v1.49 and are scaled to reflect approximately the range of variation observed in our samples. (PDF) [file pone.0085199.s006.pdf]
