# Supplementary material for: A Complex Evolutionary History in a Remote Archipelago: Phylogeography and Morphometrics of the Hawaiian Endemic Ligia Isopods
Source: PLoS One. 2013 Dec 30;8(12):e85199. doi: 10.1371/journal.pone.0085199 (PMC3875554; doi:10.1371/journal.pone.0085199)
Supplement: Table S1 — Localities included in the study, with corresponding GenBank accession numbers, and geographic information. (DOCX) [file pone.0085199.s007.docx]

**Table S1. Localities included in the study, with corresponding GenBank accession numbers, and geographic information**.

ID labels correspond with those used in the figures and text. N/A = not available.

|  |  |  | Accession Numbers | | | | | |  |  |
| --- | --- | --- | --- | --- | --- | --- | --- | --- | --- | --- |
| Species | Locality Name | ID | 16S rDNA | 12S rDNA | COI | Cyt-b | NaK | 28S rDNA | Lat. | Long. |
| *L. hawaiensis* | Waiopai, Maui | A1 | KF546549 | KF546573 |  | KF546718 |  |  | N/A | N/A |
| *L. hawaiensis* | Kealakukea Bay, Hawai'i | A2 |  | KF546574 | KF546627 |  | KF546594 |  | 19°28'32.88"N | 155°55'11.04"W |
| *L. hawaiensis* | Pu'unalu Beach Park, Hawai'i | A3 | KF546551 | KF546576 | KF546628 | KF546716 | KF546593 | KF546701 | 19° 8'0.60"N | 155°30'18.30"W |
| *L. hawaiensis* | Isaac Hale Beach Park, Hawai'i | A4 | KF546550 | KF546575 |  | KF546717 | KF546586 | KF546702 | 19°27'26.82"N | 154°50'31.68"W |
| *L. perkinsi* | Nu'uanu Pali, O'ahu | B1 | KF546548 | KF546572 | KF546661 | KF546719 |  |  | N/A | N/A |
| *L. perkinsi* | Mt Kahili, Kaua'i | C1 | KF546546 | KF546578 | KF546660 |  |  |  | N/A | N/A |
| *L. perkinsi* | Makaleha Mts, Kaua'i | C2 | KF546545 | KF546577 | KF546659 | KF546723 |  |  | N/A | N/A |
| *L. perkinsi* | Haupu Range, Kaua'i | C3 | KF546547 | KF546579 | KF546655 | KF546722 | KF546592 | KF546683-84 | N/A | N/A |
| *L. hawaiensis* | Kapua'a Beach Park, Kaua'i | D1 | KF546544 | KF546571 | KF546598-606 | KF546721 | KF546585 | KF546685-90 | 22°13'05.30"N | 159°25'31.15"W |
| *L. hawaiensis* | Kauapea Beach, Kaua'i | D2 | KF546543 | KF546570 | KF546656 | KF546720 |  |  | N/A | N/A |
| *L. hawaiensis* | Kauapea Beach, Kaua'i | D2 | AY051343 |  | AY051324 |  |  |  | N/A | N/A |
| *L. hawaiensis* | Kapa'a, Kaua'i | D3 | AY051344 |  | AY051325 |  |  |  | N/A | N/A |
| *L. hawaiensis* | Lihu'e, Kaua'i | D4 | AY051346 |  | AY051327 |  |  |  | N/A | N/A |
| *L. hawaiensis* | Kukui'ula, Kaua'i | D5 | AY051345 |  | AY051326 |  |  |  | N/A | N/A |
| *L. hawaiensis* | Ala Wai Canal, O'ahu | E1 | AY051348 |  | AY051329 |  |  |  | N/A | N/A |
| *L. hawaiensis* | Papohaku Beach Park, Moloka'i | E2 | KF546542 | KF546569 | KF546607 | KF546715 |  |  | 21°10'46.56"N | 157°15'5.88"W |
| *L. hawaiensis* | North of Puko'o, Moloka'i | E3 | KF546540 | KF546565 | KF546608-16 | KF546713 | KF546587 | KF546696-700 | 21°06'06.84"N | 156°45'06.66"W |
| *L. hawaiensis* | Manele Bay, Lana'i | E4 | KF546538 | KF546564 | KF546643-49 |  | KF546589 | KF546677-82 | 20°44'37.37"N | 156°53'12.47"W |
| *L. hawaiensis* | Poelua Bay, Maui | E5 | KF546541 | KF546566 | KF546657 | KF546711 |  |  | N/A | N/A |
| *L. hawaiensis* | Spreckelsville, Maui | E6 | KF546539 | KF546567 | KF546650-54; 95-97 | KF546712 | KF546590 | KF546691-95 | 20°54'31.38"N | 156°24'40.26"W |
| *L. hawaiensis* | Keanae, Maui | E7 | KF546537 | KF546568 | KF546658 | KF546714 |  |  | N/A | N/A |
| *L. hawaiensis* | Pupukea, O'ahu | F1 | KF546531 | KF546562 | KF546617-26 | KF546709 | KF546591 | KF546667-71 | 21°38'59.70"N | 158°03'45.48"W |
| *L. hawaiensis* | Pupukea, O'ahu | F1 | KF546533 |  |  |  |  |  | N/A | N/A |
| *L. hawaiensis* | Pupukea, O'ahu | F1 | AY051349 |  | AY051330 |  |  |  | N/A | N/A |
| *L. hawaiensis* | Pouhala Marsh, O'ahu | F2 | KF546532 |  |  | KF546710 |  |  | N/A | N/A |
| *L. hawaiensis* | Pouhala Marsh, O'ahu | F2 | AY051347 |  | AY051328 |  |  |  | N/A | N/A |
| *L. hawaiensis* | Honomanu Bay, Maui | F3 | KF546530 | KF546563 |  | KF546708 |  |  | N/A | N/A |
| *L. hawaiensis* | Keokea Beach, Hawai'i | F4 | KF546529 | KF546558 |  | KF546703 |  |  | N/A | N/A |
| *L. hawaiensis* | Onekahakaha Beach Park, Hawai'i | F5 | KF546534 | KF546561 | KF546629-42 | KF546705 | KF546588 | KF546672-76 | 19°44'16.05"N | 155°02'20.15"W |
| *L. hawaiensis* | Leleiwi Beach, Hawai'i | F6 | KF546535 | KF546560 |  | KF546706 |  |  | N/A | N/A |
| *L. hawaiensis* | South Point, Hawai'i | F7 | KF546536 | KF546559 |  | KF546707 |  |  | N/A | N/A |
| *L. hawaiensis* | Kapa'a State Park, Hawai'i | F8 | KF546528 | KF546557 |  | KF546704 |  |  | 20°12'11.52"N | 155°54'6.66"W |
| *L. exotica* | Veracruz, Mexico |  | KF546552 | KF546584 | KF546664 | KF546726 |  |  | 19°12'33.63"N | 96° 7'51.39"W |
| *L. occidentalis* | Guaymas, Mexico |  | KF546553 | KF546583 | KF546666 | KF546728 |  |  | 27°54'44.33"N | 110°56'49.56"W |
| *L. vitiensis* | Parangtritis, Java, Indonesia |  | KF546554 | KF546582 | KF546665 | KF546727 |  |  | N/A | N/A |
| *L. vitiensis* | Dili, East Timor |  | KF546556 | KF546581 | KF546662 | KF546725 |  |  | N/A | N/A |
| *L. vitiensis* | Labuanbajo, Flores, Indonesia |  | KF546555 | KF546580 | KF546663 | KF546724 |  |  | N/A | N/A |
