# Supplementary material for: A Complex Evolutionary History in a Remote Archipelago: Phylogeography and Morphometrics of the Hawaiian Endemic Ligia Isopods
Source: PLoS One. 2013 Dec 30;8(12):e85199. doi: 10.1371/journal.pone.0085199 (PMC3875554; doi:10.1371/journal.pone.0085199)
Supplement: Table S2 — Included and excluded characters and substitution models used for phylogenetic reconstructions. (DOCX) [file pone.0085199.s008.docx]

**Table S2. Included and excluded characters and substitution models used for phylogenetic reconstructions.**

| Gene | Taxa | Total Chars. | Exc. Chars. | Inc. Chars. | Pars. Inf. | AIC  (weight) | AICc  (weight) | BIC  (weight) |
| --- | --- | --- | --- | --- | --- | --- | --- | --- |
| 16S rDNA | 35 | 497 | 113 | 384 | 107 | TIM2 +I +G (0.4412) | TIM2 +I +G (0.5449) | TIM2+I+G (0.5094) |
| 12S rDNA | 28 | 510 | 106 | 404 | 120 | TIM2+I+G (0.1544) | TPM2uf+I+G (0.1767) | HKY+G (0.2746) |
| COI | 28 | 615 | 0 | 615 | 208 | 012010+I+G+F (0.2669) | 012010+I+G+F (0.3396) | 012010+I+G+F (0.6786) |
| Cyt-b | 26 | 355 | 0 | 355 | 146 | TrN+G (0.1371) | TrN+G (0.2019) | HKY+G (0.5933) |
| MT Total | 35 | 1977 | 219 | 1758 | 581 | 012313+I+G+F (0.2139) | 012313+I+G+F (0.2232) | 012010+I+G+F (0.5984) |
