# Supplementary material for: A Complex Evolutionary History in a Remote Archipelago: Phylogeography and Morphometrics of the Hawaiian Endemic Ligia Isopods
Source: PLoS One. 2013 Dec 30;8(12):e85199. doi: 10.1371/journal.pone.0085199 (PMC3875554; doi:10.1371/journal.pone.0085199)
Supplement: Table S3 — Settings for phylogenetic analyses of the concatenated mitochondrial (MT) dataset. (DOCX) [file pone.0085199.s009.docx]

**Table S3. Settings for phylogenetic analyses of the concatenated mitochondrial (MT) dataset.**

| Method | Model and Priors^1^ | Partitioning scheme^2^ | Iterations generations/ bootstrap replicates | Sample Frequency | Runs/ chains | Burn-in | ASDSF^3^ | Bayes Factors^4^/ ML Scores (-lLn) | ESS^4,5^ >200 | PSRF^6^ |
| --- | --- | --- | --- | --- | --- | --- | --- | --- | --- | --- |
| RAxML | GTR +Γ | 1 | 1,000 | n/a | n/a | n/a | n/a | -10335.851 | n/a | n/a |
| RAxML | GTR +Γ | 4 (By Gene) | 1,000 | n/a | n/a | n/a | n/a | -10261.310 | n/a | n/a |
| RAxML | GTR +Γ | 5 (PF) | 1,000 | n/a | n/a | n/a | n/a | -9701.442 | n/a | n/a |
| Garli | TIM2 +Γ | 1 | 1,000 | n/a | n/a | n/a | n/a | -10332.685 | n/a | n/a |
| Garli | Mixed Model | 4 (By Gene) | 1,000 | n/a | n/a | n/a | n/a | -10247.971 | n/a | n/a |
| Garli | Mixed Model | 5 (PF) | 1,000 | n/a | n/a | n/a | n/a | -9795.94 | n/a | n/a |
| MrBayes | GTR +Γ | 1 | 200,000,000 | 5,000 | 4 | 25% | 0.001663 | -10392.418 | Yes | 1 |
| MrBayes | GTR +Γ | 4 (By Gene) | 200,000,000 | 5,000 | 4 | 25% | 0.001071 | -10358.250 | Yes | 1 |
| MrBayes | GTR +Γ | 5 (PF) | 200,000,000 | 5,000 | 4 | 25% | 0.001462 | -10060.935 | Yes | 1 |
| Phycas | GTR +Γ | 1 | 1,000,000 | 50 | 1/1 | 25% | n/a | -10398.907 | n/a | n/a |
| Phycas | GTR +Γ | 4 (By Gene) | 1,000,000 | 50 | 1/1 | 25% | n/a | -10347.169 | n/a | n/a |
| Phycas | GTR +Γ | 5 (PF) | 1,000,000 | 50 | 1/1 | 25% | n/a | -9759.840 | n/a | n/a |

^1^ All others default; ^2^ different partitions separated by comma; ^3^ Average standard deviation of split frequencies; ^4^ estimated in Tracer v.1.5; ^5^ Effective Sample Size; ^6^ Potential Scale Reduction Factor for all parameters; PF = PartitionFinder v.1.0: (A) 12S+16S+Cytb2 (GTR +G), (B) COI1 (TrNef+I); (C) COI2+Cytb1 (F81 +I), (D) COI3 (HKY +G); (E) Cytb3 (TrN +G).
