# Supplementary material for: A Complex Evolutionary History in a Remote Archipelago: Phylogeography and Morphometrics of the Hawaiian Endemic Ligia Isopods
Source: PLoS One. 2013 Dec 30;8(12):e85199. doi: 10.1371/journal.pone.0085199 (PMC3875554; doi:10.1371/journal.pone.0085199)
Supplement: Table S4 — Support values (percentage) for phylogenetic analyses of the concatenated mitochondrial (MT) dataset. (DOCX) [file pone.0085199.s010.docx]

**Table S4: Support values (percentage) for phylogenetic analyses of the concatenated mitochondrial (MT) dataset.**

|  | RAXML | | | GARLI | | | MrBayes | | | Phycas | | |
| --- | --- | --- | --- | --- | --- | --- | --- | --- | --- | --- | --- | --- |
| Partitioning scheme | 1 | 4  (By Gene) | 5  (PF) | 1 | 4  (By Gene) | 5  (PF) | 1 | 4  (By Gene) | 5  (PF) | 1 | 4  (By Gene) | 5  (PF) |
| Model | GTR +Γ | GTR +Γ | GTR +Γ | TIM2 +Γ | Mixed Model | Mixed Model | GTR +Γ | GTR +Γ | GTR +Γ | GTR +Γ | GTR +Γ | GTR +Γ |
| Clade |  |  |  |  |  |  |  |  |  |  |  |  |
| A | 99 | 98 | 94 | 96 | 97 | 97 | 100 | 100 | 100 | 100 | 100 | 100 |
| C | 100 | 100 | 100 | 100 | 100 | 100 | 100 | 100 | 100 | 100 | 100 | 100 |
| D | 100 | 100 | 100 | 100 | 100 | 100 | 100 | 100 | 100 | 100 | 100 | 100 |
| E | 100 | 100 | 100 | 100 | 100 | 100 | 100 | 100 | 100 | 100 | 100 | 100 |
| F | 97 | 99 | 94 | 96 | 98 | 93 | 100 | 100 | 100 | 100 | 100 | 100 |
| Hawai'i^1^ | 81 | 82 | 91 | 81 | 81 | 90 | 97 | 99 | 98 | 89 | 98 | 99 |
| O'ahu^1^ | 86 | 86 | 76 | 80 | 75 | 63 | 99 | 99 | 100 | 97 | 96 | 91 |
| E+F | <50 | 56 | 58 | <50 | 55 | 52 | <50 | 65 | 64 | <50 | 69 | 62 |
| D+E+F | 76 | 78 | 69 | 74 | 75 | 64 | 98 | 99 | 87 | 98 | 99 | 98 |
| C+D+E+F | 64 | 69 | 63 | 62 | 70 | 60 | 95 | 97 | 78 | 96 | 98 | 96 |
| B+C+D+E+F | <50 | 51 | <50 | <50 | 55 | <50 | 87 | 88 | 52 | 83 | 66 | 57 |
| Ingroup | 100 | 100 | 100 | 100 | 100 | 100 | 100 | 100 | 100 | 100 | 100 | 100 |

^1^ subclade within clade F. PF = as indicated by PartitionFinder v.1.0; see Table S3.
